# Ni(II) Complexes with Mixed Ligands, Reduced N_2_O_2_ Schiff Bases and β-Diketones: Redox Modulation, ROS Generation and Antiproliferative Activity in Cancer Cells Associated with Caspase-3

**DOI:** 10.3390/ijms27156601

**Published:** 2026-07-24

**Authors:** Erika Lorena Cedillo-Gutiérrez, Adrián Espinoza-Guillén, Luis Felipe Hernández-Ayala, Esther Reveles-Ayala, Marcos Flores-Álamo, Luis Antonio Ortiz-Frade, Carmen Mejía, Lena Ruiz-Azuara

**Affiliations:** 1 Facultad de Química, Universidad Nacional Autónoma de México, Av. Universidad 3000, Circuito Exterior s/n, CU, Ciudad de México C.P. 04510, Mexicohdz.ayala@yahoo.com.mx (L.F.H.-A.); 2 Laboratorio de Biomedicina Interdisciplinaria, Centro Interdisciplinario de Biociencias Aplicadas a la Salud, Facultad de Ciencias Naturales, Universidad Autónoma de Querétaro, Santiago de Querétaro C.P. 76230, Mexico; 3 Edificio H Mario Molina, Circuito Mario de la Cueva, Esquina Circuito de la Investigación Científica, CU, Ciudad de México C.P. 04510, Mexico; 4 Centro de Investigación y Desarrollo Tecnológico en Electroquímica (CIDETEQ), Parque Tecnológico Quéretaro s/n, Sanfandila, Pedro Escobedo C.P. 76703, Mexico

**Keywords:** nickel(II), mixed chelate complexes, Hydrogenated Schiff bases, β-diketones, fluorinated ligands, redox potential, antiproliferative activity, apoptosis

## Abstract

This study addresses the development of nickel(II) complexes with potential antiproliferative activity. Two novel hydrogenated Schiff base ligands (N_2_O_2_-type), **L1** and **L2**, were synthesized using environmentally friendly routes within a green chemistry framework and fully characterized. These ligands were coordinated to Ni(II) to obtain mixed octahedral complexes of general formulae [Ni(N_2_O_2_)(NO_3_)_2_] and [Ni(N_2_O_2_)(O–O)]NO_3_, where O–O denotes β-diketones (acetylacetonate and fluorinated analogs). Structural, spectroscopic, and electrochemical characterization, including cyclic voltammetry, was performed to evaluate the effect of ligand substitution on redox properties. Antiproliferative activity was assessed in HeLa cells. The results show that all complexes exhibit octahedral geometry, while [Ni(N_2_O_2_)(O–O)]NO_3_ complexes behave as 1:1 electrolytes, with redox potentials influenced by electron-withdrawing fluorinated substituents on the secondary ligand. Complexes containing fluorinated diketones and the methoxy-substituted **L1** ligand displayed enhanced antiproliferative effects compared to non-fluorinated and unsubstituted analogues. Mechanistic studies suggest apoptosis induction associated with early caspase-3 activation, likely mediated by reactive oxygen species. Overall, ligand electronic effects play a key role in modulating redox behavior and biological activity in these nickel(II) complexes.

## 1. Introduction

In the last three decades, the design, synthesis, characterization and evaluation of new inorganic compounds against diseases such as cancer [1,2,3], diabetes [4], bacterial, viral and parasitic diseases [5,6,7,8] have been a priority for the area of Medicinal Inorganic Chemistry, which is an important branch within Bioinorganic Chemistry [9,10]. Compounds with transition metals (coordination compounds) are a very important field in this area, because metal ions can present activity in diverse biological processes such as the stabilization of protein structures, as catalysts in a great variety of enzymatic processes and actively participate in oxidation-reduction processes, as well as their capacity to initiate, modulate or inhibit reactions within biological systems [11]. Other parameters that characterize these types of systems are the variations in the geometry they adopt, depending on the ligands that are attached to them. Regarding the ligands, those can be varied, allowing for the modulation of various descriptors such as the volume and redox potential of the molecules under study. Redox potential has been observed to have a significant influence on the inhibition of proliferation in various biological systems, such as in various types of tumor cells, as well as in parasites and bacteria [7,8,11,12].

Nickel is an essential trace element in several microorganisms. Although it is not a metal as well-known as iron or zinc, it plays an important role in several biological processes. Nickel has a function as a cofactor in some enzymes, such as urease, which is essential in the nitrogen cycle [13,14]. It also participates in the biosynthesis of metals for the formation of metallic structures in proteins, and in some organisms, it is vital for iron metabolism, promoting its correct absorption and effective use [15]. On the other hand, reports of antiproliferative activity in cancer cells of nickel compounds have been found. This inhibition suggests a potential antitumor effect, where these nickel compounds can affect cell signaling promoting apoptosis (programmed cell death) in cancer cells [16,17]. Proposed mechanisms of action include the alteration of the signaling pathways that regulate the cell cycle, as well as the induction of oxidative stress, favoring cell death in tumors. Some nickel compounds have also been reported to cause DNA damage in cancer cells [18,19]. A specific study in breast cancer cell lines found that certain nickel compounds exhibit antiproliferative properties [17].

As already mentioned, ligands play a very important role in the formation of new coordination compounds. This is the case with Schiff bases and their hydrogenated derivatives, which exhibit a strong chelating effect, such as tetradentate (N_2_O_2_) ligands, in addition to their diverse biological properties such as antimicrobial, anticancer, antioxidant, and anti-inflammatory properties [20,21,22]. Metal compounds containing Schiff bases and their hydrogenated derivatives are highly versatile in coordination chemistry. Numerous syntheses of these compounds have been reported using various amines and 5R-salicylaldehyde precursors, enabling modulation of their electronic and steric properties through the incorporation of electron-withdrawing or electron-donating substituents at the 5-position of the salicylaldehyde ring [23,24,25]. Such substituent effects may alter the redox behavior, coordination geometry, and electronic distribution of the metal center, which are key factors governing their biological properties. In this context, tuning the ligand electronic environment can influence intracellular ROS production and oxidative stress pathways, potentially enhancing antiproliferative activity in cancer cell models [26,27,28].

Based on these considerations, this work proposes the synthesis and characterization of a new series of mixed nickel(II) coordination compounds containing hydrogenated Schiff base (N_2_O_2_) as the main ligand β-diketones (acac = acetylacetonate) with fluorinated substituents (acac, tfac, hfac) as a secondary ligand (Scheme 1). The incorporation of fluorinated β-diketones is expected to modulate the electronic density around the metal center through their strong electron-withdrawing character, potentially influencing the redox behavior, lipophilicity, stability, and biological activity of the resulting complexes. In particular, fluorinated ligands have been associated with enhanced membrane permeability and altered intracellular interactions, which may contribute to improved antiproliferative effects [29,30].

Additionally, the hydrogenated Schiff base framework contains ether linkages and methoxy substituents that may increase ligand flexibility and electronic delocalization while also modifying steric and donor properties around the nickel center. The presence of these donor groups may influence coordination geometry, redox potential, and reactive oxygen species (ROS) generation, parameters that are closely related to oxidative stress-mediated cytotoxic mechanisms in cancer cells. Therefore, the structural modulation achieved through both fluorinated β-diketonates and substituted (N_2_O_2_) ligands could directly impact the in vitro antiproliferative activity of the complexes against HeLa cervical cancer cells, evaluated through IC_50_ determination.

## 2. Results and Discussion

### 2.1. Synthesis and Structural Characterization of Hydrogenated Schiff Base Ligands (***L1*** and ***L2***)

For the synthesis of tetradentate hydrogenated Schiff base ligands (N_2_O_2_), an optimized synthetic route was developed involving the condensation of the corresponding primary amines with aromatic aldehydes under solvent-free conditions, followed by reduction of the resulting imine bonds using sodium borohydride (Scheme 2).

The target hydrogenated N_2_O_2_ ligands, 2,2′-(ethane-1,2-diylbis(oxy))bis(N-(4-methoxybenzyl)ethane-1-amine) (**L1**) and 2,2′-(ethane-1,2-diylbis(oxy))bis(N-benzylethan-1-amine) (**L2**), were obtained as pale-yellow oils following reduction of the corresponding Schiff base precursors. After completion of the reaction, the crude products were purified by liquid–liquid extraction to remove inorganic salts and residual reducing agent prior to structural characterization.

The structures of both ligands were established by a combination of FTIR, ^1^H and ^13^C NMR, elemental analysis, and FAB^+^ mass spectrometry. The FTIR spectra provided clear evidence for the successful reduction of the imine precursors. Both ligands exhibited broad absorptions assigned to ν(N–H) stretching vibrations of secondary amines at 3374 cm^−1^ (**L1**) and 3363 cm^−1^ (**L2**), together with N–H deformation bands at 1511 and 1500 cm^−1^, respectively. Importantly, the characteristic azomethine ν(C=N) stretching band expected for the corresponding Schiff base intermediates in the 1630–1660 cm^−1^ region was no longer observed, indicating complete reduction of the imine functionality. Aromatic ν(C=C) stretching vibrations appeared at 1610 cm^−1^ for **L1** and 1606 cm^−1^ for **L2**, whereas the ether bridge was identified by the ν(C–O–C) stretching band near 1116 cm^−1^ in both ligands. The methoxy-substituted ligand **L1** additionally displayed characteristic absorptions at 1248 and 1373 cm^−1^, assigned to the aryl–OCH_3_ and methyl group vibrations, respectively, which are absent in **L2**. Furthermore, the bands observed at approximately 822–824 cm^−1^ correspond to aromatic C–H out-of-plane bending vibrations characteristic of *para*-substituted (**L1**) and monosubstituted (**L2**) benzene rings. Taken together, these FTIR features provide strong evidence for the conversion of the Schiff base intermediates into the corresponding hydrogenated secondary amine ligands. The complete FTIR spectra are included in the Supplementary Materials (Figures S1 and S2).

The ^1^H NMR spectra were in excellent agreement with the proposed molecular structures. Signal assignments were established considering chemical shifts, multiplicities, integration values, and the molecular symmetry of both ligands. For **L1**, the aromatic region exhibited the characteristic AA′BB′ spin system expected for *para*-substituted anisole rings, giving rise to two sets of doublets centered at δ 7.20–7.24 and 6.81–6.86 ppm. This well-resolved pattern reflects the magnetic equivalence generated by the *para* substitution and confirms the high symmetry of the aromatic environment. Moreover, the upfield resonance of the protons *ortho* to the methoxy substituent results from the electron-donating (+M) effect of the methoxy group, which increases the electron density over the aromatic ring and partially shields the neighboring nuclei. A singlet at δ 3.77 ppm was readily assigned to the methoxy groups. The resonances observed between δ 3.58 and 3.71 ppm arise from overlapping signals corresponding to the benzylic methylene groups attached to nitrogen atoms (NCH_2_Ar) and to two magnetically similar oxyethylene methylene environments (OCH_2_) within the N_2_O_2_ backbone. Although the corresponding carbon atoms are resolved as two distinct resonances in the ^13^C NMR spectrum (δ 70.71 and 70.40 ppm), their proton resonances overlap because of their very similar electronic environments, giving rise to a single broad multiplet in the ^1^H NMR spectrum. The aminoethyl methylene groups adjacent to the secondary amine nitrogen atoms appeared as triplets centered at δ 2.77 ppm, in agreement with their greater shielding relative to the oxygen-bound methylene groups and with the flexibility of the aminoether backbone.

In contrast, **L2** displayed the expected aromatic multiplet centered at approximately δ 7.27 ppm, characteristic of monosubstituted benzyl rings. Unlike **L1**, the absence of the electron-donating methoxy substituent results in a more homogeneous electronic distribution over the phenyl ring, causing overlap of the aromatic proton resonances into the typical multiplet observed for benzyl derivatives. As expected, no methoxy resonance was detected in the aliphatic region. The remaining methylene resonances appeared within the same δ 2.7–3.8 ppm interval observed for **L1**, indicating that substitution on the aromatic ring has only a minor influence on the electronic environment of the N_2_O_2_ aminoether framework. Consequently, the ^1^H NMR spectra demonstrate that both ligands possess the same hydrogenated tetradentate backbone and differ only in the substitution pattern of the aromatic rings.

The ^13^C NMR spectra further corroborated the proposed structures. Ligand **L1** exhibited a characteristic resonance at δ 158.8 ppm assigned to the ipso aromatic carbon bonded to the methoxy substituent (Ar–OCH_3_). This pronounced downfield shift is characteristic of anisole derivatives and arises from the direct attachment of the aromatic carbon to the electronegative oxygen atom, which modifies the electronic distribution through resonance despite the electron-donating character of the methoxy substituent. The remaining aromatic carbon resonances appeared at δ 131.5, 126.6, and 113.8 ppm, in agreement with the expected *para*-methoxybenzyl fragment. In contrast, **L2** displayed aromatic carbon signals at δ 140.1, 128.4, 128.2, and 127.0 ppm, consistent with an unsubstituted benzyl ring lacking the resonance effects introduced by the methoxy group.

For both ligands, the oxyethylene carbons resonated at δ 70–71 ppm, as expected for methylene groups directly bonded to oxygen atoms. In comparison, the benzylic and aminoethyl methylene carbons appeared between δ 48 and 55 ppm, reflecting the electronic influence of the neighboring secondary amine nitrogen atoms. The relatively narrow chemical shift range observed for these aliphatic carbons indicates that reduction of the imine bonds preserves a chemically homogeneous and flexible aminoether framework.

At first glance, the number of observed ^13^C resonances appears lower than the total number of carbon atoms present in the molecular formulas. However, this observation is fully consistent with the high molecular symmetry of both ligands, which renders several carbon atoms chemically equivalent and therefore magnetically indistinguishable in the ^13^C NMR spectra. Consequently, only the unique carbon environments are experimentally observed, and the number of resonances agrees precisely with the symmetry expected for the proposed N_2_O_2_ aminoether structures. This behavior is characteristic of highly symmetric tetradentate ligands and further supports the assigned molecular structures. The complete signal assignments are summarized in the Experimental Section, and the corresponding ^1^H and ^13^C NMR spectra are provided in the Supplementary Materials (Figures S3–S6).

Further structural confirmation was provided by FAB^+^ mass spectrometry. Ligand **L1** exhibited a protonated molecular ion at *m*/*z* 389 corresponding to [C_22_H_32_N_2_O_4_ + H]^+^, whereas **L2** displayed a protonated molecular ion at *m*/*z* 329 assigned to [C_20_H_28_N_2_O_2_ + H]^+^. These molecular ions agree well with the expected molecular masses of the target ligands. The fragmentation pattern of **L1** showed ions at *m*/*z* 359, 281, 267, and 228, together with a base peak at *m*/*z* 122, which is reasonably assigned to a resonance-stabilized methoxybenzyl fragment generated by cleavage of the benzylic C–N bond. The enhanced stability of this fragment is consistent with delocalization of the positive charge through the anisole ring. Conversely, **L2** displayed fragment ions at *m*/*z* 239, 196, and 134, while the base peak at *m*/*z* 93 corresponds to the characteristic benzyl/tropylium cation, whose exceptional abundance arises from the aromatic stabilization of the tropylium ion. The 60 Da difference between the protonated molecular ions of **L1** and exactly matches the mass contribution of the two methoxy substituents present in **L1**, providing additional evidence that both ligands possess the same hydrogenated N_2_O_2_ framework and differ only in the substitution pattern of the aromatic rings. The corresponding FAB^+^ mass spectra are provided in the Supplementary Materials (Figures S7 and S8).

Overall, the combined FTIR, ^1^H and ^13^C NMR, elemental analysis, and FAB^+^ mass spectrometric results provide consistent and complementary evidence supporting the successful synthesis of ligands **L1** and **L2**. Beyond confirming the molecular composition, the spectroscopic data reveal the electronic effects exerted by the *para*-methoxy substituents, demonstrate preservation of the symmetric N_2_O_2_ aminoether backbone following reduction of the Schiff base intermediates, and explain the observed differences between **L1** and **L2** in terms of their aromatic substitution patterns. Collectively, these results confirm the successful preparation of the proposed hydrogenated tetradentate ligands, which constitute a suitable platform for the synthesis and subsequent physicochemical, electrochemical, theoretical, and biological evaluation of the corresponding Ni(II) mixed-ligand complexes.

### 2.2. X-Ray Structure Analysis of ***L1***

Single-crystal X-ray diffraction analysis reveals that **L1** crystallizes as a discrete ionic species formed by the cation 2,2′-(ethane-1,2-diylbis(oxy))bis(N-(4-methoxybenzyl)ethane-1-aminium) balanced by two nitrate counter anions (Figure 1). Since the hydrogenated ligand was obtained as a viscous liquid, additional crystallization experiments were carried out to isolate the compound in a crystalline form suitable for structural characterization. For this purpose, nitric acid was employed to protonate the ligand, leading to the formation of the corresponding nitrate salt. Consequently, nitrate anions were incorporated as charge-balancing counterions in the final crystalline species. The asymmetric unit contains one half of the organic cation and one nitrate anion, indicating that the complete molecular entity is generated by crystallographic symmetry. This arrangement is consistent with the centrosymmetric nature of the crystal lattice and reflects an efficient packing of the charged species. The molecular geometry of the cation shows a clear deviation from planarity between the aliphatic ammonium fragment and the aromatic ring. Specifically, the plane defined by atoms N(1)–C(7)–C(8)–C(9) forms a dihedral angle of 63.5(2)° with the aromatic ring, evidencing a significant torsional distortion. This non-coplanarity is further corroborated by the torsion angles C(2)–C(1)–C(7)–N(1) [106.9(10)°] and C(6)–C(1)–C(7)–N(1) [73.1(12)°], which correspond to the rotational freedom around the C(1)–C(7) bond and the steric demands imposed by the substituents. Such conformational flexibility is commonly observed in protonated ammonium derivatives and can play an important role in accommodating intermolecular interactions in the solid state. In contrast, the methoxy substituent is essentially coplanar with the aromatic ring, as indicated by a very low root-mean-square deviation (rms = 0.0120 Å). This planarity favors effective conjugation between the oxygen lone pairs and the π-system of the phenyl ring. Consistently, the O(1)–C(4) bond [1.379(5) Å] is shorter than the O(1)–C(11) bond [1.416(6) Å], reflecting partial double-bond character arising from π-delocalization. Such bond length differentiation is a well-established structural signature of anisotropic conjugation in aromatic methoxy groups. All observed bond lengths and angles fall within expected ranges for related organic ammonium salts (Table 1). Notably, the N(1)–C(7) [1.499(2) Å] and N(1)–C(8) [1.490(2) Å] distances are characteristic of single N–C bonds involving an sp^3^-hybridized nitrogen atom. Together with the tetrahedral geometry around N(1), these parameters unambiguously confirm the protonated nature of the nitrogen center. This structural feature clearly distinguishes **L1** from analogous Schiff base compounds reported in the literature, in which imine N=C double bonds are present [31]. The absence of such a double bond in **L1** is consequence of the protonation and saturation of the nitrogen atom, which has important implications for the ligand’s electronic properties and potential coordination behavior. The ether bridge within the aliphatic backbone displays nearly equivalent C–O bond lengths [O(2)–C(9) = 1.421(2) Å and O(2)–C(10) = 1.4204(19) Å], as expected for non-conjugated single C–O bonds. This equivalence indicates a uniform electronic environment around the ether oxygen, in contrast to the differentiated bonding observed in the aromatic methoxy group.

Analysis of crystal packing reveals that the stability of the structure in the solid state is mainly constituted by intermolecular interactions N–H···O hydrogen bonds between the protonated ammonium groups and the nitrate anions. Two significant interactions, N(1)–H(1E)…O(5) and N(1)–H(1D)…O(4), related by the symmetry operation (x, 3/2 − y, −1/2 + z), organize the ions into extended supramolecular motifs. These interactions generate R_4_^4^(30) ring motifs and C_4_^4^(30) chain motifs that propagate along the a–b plane, giving rise to a two-dimensional hydrogen-bonded network. The cooperative nature of these hydrogen bonds not only neutralizes the electrostatic charges of the ionic components but also enforces a well-defined packing arrangement, as illustrated in Figure 2. Such hydrogen-bond-driven assemblies are typical for organic ammonium nitrates and play a decisive role in determining crystal cohesion, thermal stability, and potentially the material’s physicochemical properties.

### 2.3. Synthesis and Structural Characterization of Coordination Compounds of Ni(II)

A series of new nickel(II) coordination compounds were synthesized and characterized using hydrogenated Schiff base ligands as primary ligands (**1a**, **1b**), along with mixed-ligand systems incorporating acetylacetonate derivatives, both non-fluorinated and fluorinated, as secondary ligands (**2a**, **3a**, **4a**, **2b**, **3b**, **4b**) (Scheme 3).

The FT-IR spectra display characteristic bands associated with ligands **L1** and **L2**, showing variations in intensity and shifts upon coordination to the metal center. The appearance of bands assigned to carbonyl C=O stretching and C–F vibrations of the diketone moieties provides clear evidence for the formation of mixed-ligand complexes. Notably, the presence of fluorine substituents in the diketones induces a shift in the carbonyl stretching bands toward higher wavenumbers, reflecting their electron-withdrawing effect. Bands observed around 1380 cm^−1^ are attributed to the nitrate (NO_3_^−^) group acting as a counterion in the mixed compounds. In contrast, for compounds **1a** and **1b**, the nitrate is coordinated to the metal center, as indicated by the characteristic splitting (doublet) in the 1300–1303 cm^−1^ region [32,33]. The main FT-IR data are summarized in Table 2, while the complete spectra are provided in the Supplementary Materials (Figures S9–S16).

Molar conductivity measurements indicate that compounds **1a** and **1b** behave as neutral species, whereas the mixed-ligand complexes (**2a**, **3a**, **4a**, **2b**, **3b**, **4b**) exhibit conductivity values consistent with 1:1 electrolyte [34]. These results support the assignment of nitrate ions as counterions in the mixed systems.

Magnetic susceptibility measurements indicate that all compounds exhibit paramagnetic behavior, consistent with the presence of two unpaired electrons and a d^8^ electronic configuration for the Ni(II) center, suggesting an octahedral geometry. This assignment is further supported by diffuse reflectance electronic spectra, in which the three characteristic transitions of Ni^2+^ (3d^8^) complexes in an octahedral ligand field are observed (Table 3), with a ^3^A_2_g ground state [35,36]. These d–d transitions arise from electron promotion between crystal field-split d orbitals and are spin-allowed but Laporte-forbidden [37]. The corresponding diffuse reflectance spectra are provided in the Supplementary Materials (Figures S17–S24).

Electronic absorption spectra recorded in CH_3_CN solution display similar profiles in the UV–Vis region (205–386 nm), indicating that structural variations among the complexes do not significantly affect the main chromophore system. In all cases, two intense absorption bands are observed in the ranges of 205–226 nm and 256–274 nm, which are assigned to π→π* transitions localized on the aromatic rings of ligands **L1** and **L2**. These bands exhibit high molar extinction coefficients, characteristic of electronically allowed transitions, and are consistent with those reported for conjugated aromatic systems (Table 3) [38,39].

A third band of lower energy appears in the 302–317 nm region and is attributed to π→π* transitions associated with the conjugated β-diketone ligand (Table 3). This behavior is consistent with electron delocalization in the enolic form of the ligand, which enhances conjugation and results in a bathochromic shift [37,39].

A more detailed comparison reveals subtle electronic effects associated with ligand substitution. Complexes derived from **L1** (**2a**, **3a**, **4a**) exhibit a slight bathochromic shift relative to their **L2** (**2b**, **3b**, **4b**) analogues, which can be attributed to the presence of the electron-donating methoxy group (Table 3). This substituent increases electron density over the aromatic system, stabilizing the excited states and lowering the energy of the π→π* transitions. In contrast, complexes containing fluorinated β-diketone ligands tend to show slight hypsochromic shifts in the corresponding bands, reflecting the strong electron-withdrawing character of fluorine, which reduces electron density within the conjugated system and increases transition energies. These observations are consistent with the expected electronic effects of substituents in conjugated systems and agree with previous reports [37,38,39].

For compounds **1a** and **1b**, an additional weak band is observed at approximately 383 and 386 nm, respectively (Table 3). This absorption is detected only in the nitrate complexes and occurs at a lower energy than the π→π* transitions, so it could be tentatively assigned to a charge-transfer (CT) transition involving the Ni(II) coordination sphere and the coordinated ligands. The low intensity of this band suggests poor orbital overlap and/or a mixed ligand-field character, rather than a fully allowed charge-transfer process [40,41]. The absence of this band in the diketonate complexes indicates that nitrate substitution alters the electronic structure, diminishing or masking this band.

Overall, the spectroscopic results indicate that the UV region is dominated by intraligand (π→π*) transitions, while the near-visible region is mainly influenced by charge-transfer contributions. The observed modulation of the absorption bands reflects the interplay between electron-donating and electron-withdrawing substituents, which fine-tune the electronic density across the ligand framework and the metal center. This behavior is characteristic of Ni(II) complexes with conjugated organic ligands and oxygenated anionic groups [37,38], and is consistent with the trends observed in the electrochemical analysis. The complete UV–Vis spectra are included in the Supplementary Materials (Figures S25–S34).

To support the assignment of signals in the electronic spectra, TD-DFT calculations were performed for all Ni(II) complexes at the UB3LYP/LANL2DZ(Ni)/6-311 + G(d,p)/SMD(CH_3_CN) level of theory. The simulated spectra reproduce the main experimental features in the UV region, particularly the intense absorptions assigned to ligand-centered π→π* transitions. For **L1**-containing complexes, the associated calculated bands appear close to the experimental absorptions at 225–226 nm and 273–274 nm, while for **L2** analogs, the corresponding calculated transitions reproduce the higher-energy experimental bands at 205–207 nm and 256–257 nm. Furthermore, the lower-energy UV bands observed for the mixed β-diketonate complexes in the 306–316 nm region are reasonably well reproduced by calculations, supporting their classification as π→π* transitions with a contribution from the conjugated β-diketonate fragment.

The weak absorptions observed experimentally in the near-visible region for **1a** and **1b** are also qualitatively supported by calculated low-intensity transitions in the 385–411 nm range. These transitions could involve a mixed ligand-field and charge-transfer character. In contrast, the lower-energy d-d bands observed by diffuse reflectance spectroscopy in the NIR region are only qualitatively reproduced. This discrepancy is expected, since ligand field transitions in open-shell Ni(II) complexes are weak, highly sensitive to the coordination environment and solid-state effects, and are often less accurately described by conventional TD-DFT. Overall, the calculations support experimental observations that the UV region is dominated by ligand-centered π→π* transitions, while the visible and lower-energy NIR features arise mainly from weak ligand field and charge-transfer contributions. The calculated spectra and the experimental-theoretical comparison are provided in Table S1 and Figures S35 and S36 of the Supporting Information.

In order to clarify the electronic nature of the weak absorptions (CT and d-d) observed in the complexes, a natural transition orbital (NTO) analysis was performed for the corresponding TD-DFT transitions. Figure 3 shows the NTOs for the CT (λ_exp_ = 383 nm, λ_calc_= 385 nm) for the **1b** complex and the respective NTOs for the absorption observed at (λ_exp_ = 674 nm, λ_calc_ = 734 nm) for **2a**–**4a** compounds.

The NTO analysis of nitrate complex shows that the hole orbital is primarily located over the tetradentate ligand structure, while the particle orbital is predominantly centered on the Ni center, with an additional contribution from the axial coordination sphere region. This result supports the classification of this weak band as a transition with a primarily ligand-metal charge-transfer (LMCT) character, accompanied by some ligand field mixing.

On the other hand, the calculated NTOs for the low-energy transition of complex **2a** show that both the hole orbital and the particle orbital are primarily localized within the Ni(II) coordination sphere, with appreciable delocalization over the donor atoms of the surrounding ligands. The inclusion of fluorinated substituents in the compounds **3a** and **4a** increases this delocalization around the dicetonate. This pattern is consistent with a mixed d-d/ligand field transition. The remain compounds (**1a** and **2b**–**4b**) follow the same trend and the NTOs are depicted in the Figure S37.

### 2.4. Electrochemistry Study

The electrochemical behavior of the Ni(II) complexes was investigated by cyclic voltammetry using 2.0 mM complex solutions in acetonitrile (CH_3_CN). Tetrabutylammonium hexafluorophosphate (TBAPF_6_) 0.1 M was used as the supporting electrolyte. Prior to each measurement, the working electrode was mechanically polished with diamond powder. The solutions were purged with nitrogen for 10 min to remove dissolved oxygen. All potentials were internally referenced to the ferrocenium/ferrocene (Fc^+^/Fc) redox couple, following IUPAC recommendations for non-aqueous systems [42]. Ohmic drop (iR) compensation was applied using a positive feedback method.

Cyclic voltammograms of the Ni(II) complexes exhibit similar overall profiles (Figure 4), consisting of one cathodic (Ic) and two anodic (Ia and IIa) processes. Representative voltammograms for all complexes are provided in the Supplementary Materials (Figures S35–S42).

The anodic process IIa is irreversible and is tentatively assigned to an oxidation process involving the coordinated complex. Although the present electrochemical data do not allow an unambiguous assignment of the electroactive center, the systematic shifts observed upon substitution of nitrate by β-diketonate ligands indicate that the oxidation response is significantly modulated by the electronic properties of the coordinated ligands [42,43]. Replacement of coordinated nitrate by β-diketonate ligands, particularly fluorinated derivatives, produces systematic changes in both the FT-IR and UV−Vis spectra. FT-IR spectra show a shift in the ν(C=O) stretching bands toward higher wavenumbers, consistent with the electron-withdrawing character of the fluorinated substituents. Likewise, UV−Vis spectra exhibit hypsochromic shifts and slightly higher transition energies compared with the nitrate analogues, indicating modification of the ligand field and redistribution of electron density around the Ni(II) center. These spectroscopic observations are consistent with the anodic potential shifts observed in cyclic voltammetry, suggesting that the oxidation process is strongly influenced by ligand electronic effects.

The Ia/Ic couple exhibits quasi-reversible behavior, with ΔEp values ranging from 73 to 102 mV and Ipa/Ipc ratios between 0.65 and 1.01 at a scan rate of 300 mV s^−1^ (Table 4). These parameters deviate from those expected for a fully reversible one-electron process, indicating moderately slow electron-transfer kinetics and/or the occurrence of a coupled chemical step (EC mechanism) [44]. Consequently, anodic (Epa) and cathodic (Epc) peak potentials provide a more appropriate description of the electrochemical behavior than formal half-wave potentials. Although the relatively high scan rate contributes to increased peak separation, the observed electrochemical parameters indicate that electron transfer is not fully reversible under the present experimental conditions.

The redox response is strongly dependent on the coordination environment around the nickel center. The hydrogenated Schiff base ligands act as tetradentate N_2_O_2_ donors, forming octahedral mixed-ligand complexes upon coordination of either nitrate or β-diketonate ligands. Complexes of series a, containing methoxy substituents on the aromatic rings, consistently exhibit more negative cathodic peak potentials (Epc) than the corresponding unsubstituted analogues of series b, indicating that the electron-donating methoxy groups increase the electron density at the nickel center and render reduction less favorable.

In contrast, replacement of coordinated nitrate by β-diketonate ligands, particularly fluorinated derivatives, shifts the cathodic peak potentials toward less negative values, reflecting the electron-withdrawing character of these ligands and the consequent stabilization of the reduced state. Based on the electrochemical profiles and previous reports for structurally related nickel systems [40], the Ic/Ia couple is tentatively assigned to the Ni(II)/Ni(I) redox process. Overall, the relatively narrow ΔEp range across the series suggests comparable electron-transfer kinetics, whereas the systematic variations in Epc demonstrate that both the donor ability of the tetradentate Schiff base and the electron-withdrawing character of the coordinated β-diketonates effectively modulate the redox properties of these Ni(II) complexes [45,46].

### 2.5. Biological Activity

The cytotoxic activity of the nickel coordination compounds and free ligands was evaluated in HeLa tumor cells using the sulforhodamine B (SRB) assay [47]. This colorimetric method quantifies total cellular protein content as an indirect measure of cell viability, allowing discrimination between metabolically active and non-viable cells, and enabling the assessment of cell growth inhibition in response to the tested compounds.

A total of eight coordination compounds (**1a**, **2a**, **3a**, **4a**, **1b**, **2b**, **3b** and **4b**), together with the free ligands (**L1** and **L2**), were evaluated. Serial dilutions were prepared from a 200 μM stock solution to obtain final concentrations of 200, 150, 100, 50, and 10 μM. The compounds were added to 96-well plates containing previously seeded HeLa cells (2 × 10^4^ cells per well). Under these experimental conditions, measurable inhibition of cell proliferation was observed, particularly at concentrations ≥100 μM.

The IC_50_ values are summarized in Table 5 and are expressed as the mean ± standard deviation (SD) of three independent biological experiments. Among the analyzed compounds, the “a” series (**1a**–**4a**) exhibited moderate cytotoxic activity, with IC_50_ values ranging from 56.0 to 139.7 μM. Notably, compound 4a displayed the highest antiproliferative activity (IC_50_ = 56.0 ± 8.2 μM), followed by compounds **3a** and **2a**. In contrast, all compounds belonging to the “b” series (**1b**–**4b**), as well as the free ligands (**L1** and **L2**), showed negligible cytotoxicity (IC_50_ > 200 μM) under the experimental conditions. For transparency, the individual IC_50_ values obtained from each independent experiment, together with the corresponding coefficients of determination (R^2^) for the dose–response fits and the calculated mean ± SD for each compound, are provided in Table S2 of the Supplementary Materials.

These results suggest that the coordination environment and structural features present in the “a” series play a crucial role in modulating biological activity. The absence of activity in the free ligands indicates that metal coordination is required to induce antiproliferative effects and may contribute to changes in physicochemical properties such as electronic distribution, molecular stability, redox behavior, and cellular uptake.

Previous studies on Ni(II) coordination compounds have demonstrated that antiproliferative activity frequently depends not only on the metal center but also on ligand-induced modulation of electronic structure, lipophilicity, and coordination environment [48,49]. In particular, β-diketonate substitution and the incorporation of electron-withdrawing groups have been associated with changes in redox behavior and biological response through modification of the electronic properties of the coordination sphere [50,51]. In agreement with these observations, the enhanced activity observed for **4a** appears consistent with the spectroscopic and electrochemical trends discussed above, where ligand substitution influenced both electronic distribution and redox properties.

#### 2.5.1. NiL1hfac (**4a**) Inhibits Cell Proliferation and Produces Morphological Changes

After treatment, morphological changes were observed, becoming more evident over longer periods. At 3 h, cells with rounded shapes were observed, exhibiting blebbing and the early formation of apoptotic bodies. In contrast, most cells treated for 4 h remained more rounded, consistent with programmed cell death compatible with apoptosis (Figure 5). The results strongly suggest that this compound has a significant antineoplastic effect on cervical cancer cells through its ability to inhibit cell proliferation, apparently via an apoptotic mechanism.

Metabolic drugs have emerged as a therapeutic alternative against various tumors, including cervical cancer [52,53]. The effects of these compounds can be observed at different levels, one of the earliest and most evident being changes in cell morphology. In the present study, HeLa cells treated with compound 4a lost their characteristic cellular projections and progressively adopted a rounded morphology, accompanied by membrane blebbing, a typical feature associated with apoptotic processes. Multiple studies have focused on understanding apoptosis, which is characterized by biochemical events including caspase activation, chromatin condensation, peripheral chromatin rearrangement, DNA fragmentation, cell shrinkage, and the formation of apoptotic bodies [49,54]. The morphological alterations observed here are consistent with these events and support the occurrence of programmed cell death.

Interestingly, the biological response observed for **4a** may also be interpreted considering the electronic and redox properties of the coordination complexes. Previous reports have suggested that the biological activity of transition metal complexes can be influenced by their ability to participate in electron-transfer processes and modulate intracellular oxidative balance, where the redox properties of the coordination sphere play an important role in determining cellular responses [26,55]. Since ROS production and intracellular redox balance are closely interconnected, modulation of the redox properties of coordination compounds may influence oxidative signaling, mitochondrial function, and apoptotic activation [26,55,56].

In the present work, cyclic voltammetry demonstrated ligand-dependent modulation of the electrochemical response across the nickel complexes, with variations in the Ni(II)/Ni(I) redox couple depending on ligand substitution. Although electrochemical measurements were performed in a non-biological medium (CH_3_CN/TBAPF_6_), these results suggest differences in the electronic accessibility of redox processes among the complexes. Interestingly, compound **4a**, which exhibited the highest antiproliferative activity, also presented ligand environments associated with electronic modulation in the spectroscopic and electrochemical studies. Although no direct relationship can be established from the present data, these observations raise the possibility that ligand-dependent modulation of redox properties may contribute to intracellular signaling processes associated with the observed biological response [55,56].

Our group has previously tested metal-based compounds such as Cu(II) on HeLa cells, where we observed the apoptotic phenomenon mainly via the mitochondrial pathway through the presence of caspase-3, along with the expression of reactive oxygen species (ROS) such as hydrogen peroxide (H_2_O_2_), hydroxyl radicals (•OH) and superoxide (O_2_•^−^) [53]. Although the present study does not establish a direct mechanistic relationship between electrochemical behavior and ROS generation, the combined evidence of morphological changes, early caspase-3 activation, and oxidative imbalance supports further investigation into the role of redox processes in the biological activity of compound **4a**.

#### 2.5.2. Caspase-3 Expression at Short Time Points

To confirm the presence of programmed cell death by apoptosis, we looked for the presence of caspase-3 in HeLa cells treated with **4a** using a highly sensitive test such as Dot Blot. We found that from 30 min of treatment, the cells were positive for this apoptotic protein. This presence was maintained throughout the entire time frame (30 min, 1, 2, 3, and 4 h), which led us to establish 30 min as the most appropriate time to confirm the presence of apoptosis (Figure 6).

Based on time kinetics, we established that the appearance of caspase-3 was the precise point to determine that apoptosis had occurred [57] and the precise time to carry out the following biological determinations.

Something that caught our attention was the fact that caspase-3 was found at 30 min, unlike in HeLa cells treated with Casiopeína IIgly, which also showed apoptosis but at 6 h. It is worth mentioning that the IC_50_ of the Cu(II) coordinated compound was 40 μM [53], while that of compound **4a** was higher (56.0 μM).

#### 2.5.3. Pro-Oxidant Environment Is an Effect of NiL1hfac (**4a**)

Nickel and nickel coordination compounds have been reported to promote oxidative imbalance, DNA damage, and apoptosis through redox-related processes involving endogenous cellular reducing agents as electron donors [58]. These mechanisms have been associated with antiproliferative responses in several tumor models, including pancreatic, breast, and skin cancer [59,60,61].

In addition, nickel species may interact with intracellular thiols such as glutathione (GSH), forming redox-active complexes capable of modifying cellular oxidative homeostasis [62]. Such interactions may alter ROS production and contribute to downstream signaling events involved in cell death pathways.

In the present study, treatment with **4a** altered the intracellular oxidative environment, as evidenced by changes in superoxide levels detected after 30 min of exposure (Figure 7). Since oxidative stress is recognized as an important regulator of apoptosis, modulation of ROS levels may influence multiple cellular processes, including mitochondrial signaling, antioxidant defense systems, and activation of programmed cell death pathways [63]. Several mechanisms have been proposed to regulate these responses, including increased intracellular ROS generation, depletion of endogenous antioxidants, modulation of manganese superoxide dismutase (Mn-SOD), regulation of glutathione peroxidase (GPx), and altered activity of Cu/Zn-SOD, all of which contribute to maintaining intracellular redox balance and controlling apoptotic signaling [64,65,66].

Interestingly, superoxide levels measured at 30 min were lower than those observed in untreated cells. This finding suggests that the oxidative response induced by **4a** may involve compensatory antioxidant mechanisms rather than simple ROS accumulation. Possible explanations include increased antioxidant buffering by GSH, enhanced ROS scavenging activity, or regulation of antioxidant enzymes such as Cu/Zn-SOD. However, these hypotheses were not directly evaluated in the present study and therefore require additional experimental confirmation.

Taken together with the observed morphological changes and early caspase-3 activation, these results suggest that modulation of intracellular redox homeostasis may contribute to the antiproliferative activity of **4a**, although the precise molecular mechanism remains to be elucidated.

## 3. Materials and Methods

### 3.1. Materials and Reagents

All chemicals were purchased from Sigma-Aldrich^®^ and were used without further purification. Solvents were dried before use by distillation over magnesium, was kept in an N_2_ atmosphere in a flask with a 3 Å molecular sieve. Synthesis of coordination compounds were carried out under nitrogen atmosphere. Schiff base-type ligands were prepared in the absence of solvent. Their hydrogenation was carried out under a nitrogen atmosphere and methanol as solvent.

### 3.2. Physical Measurements

Elemental analysis was performed on an EAGER 200 analyzer (EAGER 200 CHNS/method). The attenuated total reflectance Fourier-transform infrared (ATR-FTIR, 4000–400 cm^−1^) spectra were obtained using a PerkinElmer Spectrum 400 instrument (PerkinElmer Inc., Waltham, MA, USA). Room-temperature magnetic susceptibility measurements (25 °C) were performed using a Johnson–Matthey magnetic susceptibility balance (Johnson Matthey PLC, London, UK) calibrated with Hg[Co(SCN)_4_] as the standard. Magnetic susceptibilities were determined by the mass-susceptibility method. The experimental mass susceptibilities were converted to molar magnetic susceptibilities (χM) and corrected for diamagnetic contributions using Pascal’s constants [67]. The corrected molar susceptibilities (χM,corr) were subsequently used to calculate the effective magnetic moments (μeff) according to the relationship μeff = 2.84(χM,corrT)1/2, where T is the absolute temperature. This procedure allows the estimation of the number of unpaired electrons and provides information about the electronic configuration of the metal center. The ^1^H and ^13^C NMR spectra were acquired using VARIAN VNMRS 200 and 300 MHz spectrometers (Varian Inc., Palo Alto, CA, USA) with tetramethylsilane (TMS) as the internal standard and CDCl_3_ as the solvent. Conductivity analysis was performed using a JENWAY 4350 Conductimetry and pH meter (Jenway Ltd., Stone, Staffordshire, UK), which was previously calibrated with 1.0, 0.1 and 0.01 mol L^−1^ KCl solutions. Solutions of each compound were prepared at a concentration of 0.001 mol L^−1^ in acetonitrile at 25 °C. The electronic spectra were obtained in a GENESYS 10S THERMO SCIENTIFIC diode array spectrophotometer in a range of 100–1100 nm, using a 3 mL quartz cell. For diffuse reflectance, a CARY VARIAN instrument was used in a range of 1000 cm^−1^ to 35,000 cm^−1^. Diffuse reflectance spectra were recorded using a Cary 5000 UV–Vis–NIR spectrophotometer (Agilent Technologies, Santa Clara, CA, USA) equipped with an integrating sphere. No mathematical transformation (e.g., Kubelka–Munk) was applied to the recorded spectra. Mass spectrometric analyses were performed employing different ionization methods depending on the type of compound. The ligands were characterized by FAB^+^-MS on a JEOL AccuTOF JMS-T100LC mass spectrometer (JEOL Ltd., Akishima, Tokyo, Japan) with acetonitrile as solvent, while the coordination compounds were analyzed by DART^+^-MS using the same instrument.

### 3.3. Synthesis

#### 3.3.1. Synthesis of Tetradentate Hydrogenated Schiff Base Ligands (N_2_O_2_)

***Synthesis of 2,2′-(ethane-1,2-diylbis(oxy))bis(N-(4-methoxybenzyl)ethane-1-amine) (L1).*** 4-Methoxybenzaldehyde (0.238 mL, 2.0 mmol) was placed in a round-bottom flask and gently heated under magnetic stirring in an oil bath to remove residual moisture. Subsequently, 2,2′-(ethane-1,2-diylbis(ethylamine)) (0.171 mL, 1.2 mmol) was added dropwise under continuous stirring. The reaction mixture was allowed to react at room temperature for approximately 30 min, during which a gradual color change from colorless to yellow was observed, indicating formation of the corresponding Schiff base intermediate. The intermediate was dissolved in methanol and reduced with NaBH_4_ (0.1664 g, 4.4 mmol). After completion of the reduction, the reaction mixture was extracted with CHCl_3_/H_2_O (4 × 20 mL). The organic phase was dried over anhydrous Na_2_SO_4_, filtered, and concentrated under reduced pressure to afford **L1** as a pale-yellow viscous oil (50.6%). Elemental analysis calculated for C_22_H_32_N_2_O_4_ (388.23 g/mol): C, 68.01; H, 8.30; N, 7.21. Found: C, 66.29; H, 7.69; N, 6.85. IR (KBr, ν_max/cm^−1^): 3374 ν(N-H), 3068 ν(Ar–H), 2901, 2830 ν(C–H), 1610 ν(Ar C=C), 1511 (δN–H), 1373 (δCH_3_), 1248 ν(Ar–OCH_3_), 1116, 1032 ν(C–O–C), 822 (γAr–H). Λ_M (CH_3_OH)= 42 µS cm^−1^. UV–Vis (CH_3_CN), λ_max (ε, L mol^−1^ cm^−1^): 225 (135561), 276 (32026). FAB^+^ MS, m/z: 389 [M + H]^+^. ^1^H NMR (200 MHz, CDCl_3_) δ: 7.26 (d, J = 8.8 Hz, 4H, Ar–H), 6.87 (d, J = 8.8 Hz, 4H, Ar–H), 3.79 (s, 6H, OCH_3_), 3.72 (s, 4H, NCH_2_Ar), 3.65–3.53 (m, 8H, overlapping OCH_2_ and NCH_2_Ar resonances), 2.84–2.68 (m, 4H, NCH_2_), 2.01 (br s, 2H, NH). ^13^C NMR (50 MHz, CDCl_3_) δ: 158.74 (Ar–C(OCH_3_)), 132.50 (C_ipso–CH_2_N), 129.50 (Ar–CH), 113.89 (Ar–CH), 70.71, 70.40 (OCH_2_), 55.38 (OCH_3_), 53.41 (NCH_2_Ar), 48.70 (NCH_2_).
***Synthesis of 2,2′-(ethane-1,2-diylbis(oxy))bis(N-benzylethan-1-amine) (L2)*.** Benzaldehyde (0.202 mL, 2.0 mmol) was placed in a round-bottom flask and gently heated under magnetic stirring in an oil bath to remove residual moisture. Subsequently, 2,2′-(ethane-1,2-diylbis(ethylamine)) (0.172 mL, 1.2 mmol) was added dropwise under continuous stirring. The reaction mixture was allowed to react at room temperature for approximately 30 min, during which a gradual color change from colorless to deep yellow was observed, indicating formation of the corresponding Schiff base intermediate. The intermediate was dissolved in methanol and reduced with NaBH_4_ (0.1664 g, 4.4 mmol). After completion of the reduction, the reaction mixture was extracted with CHCl_3_/H_2_O (4 × 20 mL). The organic phase was dried over anhydrous Na_2_SO_4_, filtered, and concentrated under reduced pressure to afford **L2** as a very pale-yellow viscous oil (49.1%). Elemental analysis calculated for C_20_H_28_N_2_O_2_ (328.46 g/mol): C, 73.13; H, 8.59; N, 8.52. Found: C, 68.0; H, 8.03; N, 8.88. IR (KBr, ν_max/cm^−1^): 3363 ν(N–H), 3065 ν(Ar–H), 2906, 2872 ν(C–H), 1606 ν(Ar C=C), 1500 (δN–H), 1116 ν(C–O–C), 824 (γAr–H). Λ_M (CH_3_OH) = 36 μS cm^−1^. UV–Vis (CH_3_CN), λ_max (ε, L mol^−1^ cm^−1^): 224 (118353), 259 (56876). FAB^+^ MS, m/z: 329 [M + H]^+^. ^1^H NMR (300 MHz, CDCl_3_) δ: 7.35–7.20 (m, 10H, Ar–H), 3.79 (s, 4H, NCH_2_Ar), 3.66–3.53 (m, 8H, overlapping OCH_2_ and NCH_2_Ar resonances), 2.85–2.73 (m, 4H, NCH_2_), 1.99 (br s, 2H, NH). ^13^C NMR (75 MHz, CDCl_3_) δ: 140.27 (C_ipso–CH_2_N), 128.61, 128.44, 127.18 (Ar–CH), 70.71, 70.48 (OCH_2_), 54.05 (NCH_2_Ar), 48.84 (NCH_2_).

#### 3.3.2. Synthesis of Coordination Compounds

Complexes **1a** and **1b** were synthesized by dissolving 1 mmol of nickel(II) nitrate hexahydrate in acetonitrile, followed by the slow addition of 1 mmol of the corresponding N_2_O_2_ ligand (**L1** or **L2**) dissolved in acetonitrile. The reaction mixture was stirred under reflux for 4 h. The resulting products were precipitated by the addition of diethyl ether, affording a fine light greenish-blue powder for **1a** and a fine light sky-blue powder for **1b**.

To prepare the mixed-ligand complexes containing β-diketonate co-ligands, 1 mmol of complexes **1a** or **1b** was dissolved in acetonitrile, followed by the addition of 1 mmol of the corresponding β-diketone, previously deprotonated with 1 mmol of triethylamine. The reaction mixture was stirred under reflux for 4 h, concentrated under reduced pressure, and the products were precipitated by the addition of diethyl ether. The resulting solids were collected by filtration and thoroughly washed several times with diethyl ether, followed by small portions of cold ethanol to remove residual triethylammonium nitrate, unreacted starting materials, and other soluble impurities. Finally, the products were dried under vacuum.

**1.**  ***[Ni(L1)(NO_3_)_2_]: (Nitrate)(2,2′-(ethane-1,2-diylbis(oxy))bis(N-(4-methoxybenzyl)ethan-1-amine)nickel(II)*, *(1a).*** Yield: 61%, m.p. 124–128 °C. Elemental analysis calculated for [Ni(C_22_H_32_N_2_O_4_)(NO_3_)_2_]·(H_2_O)_2_ (607.23 g/mol): C, 43.51; H, 5.97; N, 9.22. Found: C, 43.9; H, 6.19; N, 9.10; S. IR (ATR, ν/cm^−1^): ʋ(R-NH-R)3642, ʋ(NH_flex_) 1514, ʋ(C-H_ar_) 3068, 822, ʋ(CH_2_, CH_3_) 2901, 2830, ʋ(C=C) 1463 ʋ(0N0_2_) 1303, ʋ(C-O-C) 1137, ʋ(C-O-C_ar_)_as_ 1248, ʋ(C-O-C_ar_)_sym_ 1032. Λ_M (CH_3_CN) = 61.1 µS cm^−1^. µ*eef* (BM) = 3.4054. MS, *m*/*z* = 508 (35%). Electrochemical data: E_½_^I^ = −1.0862 V (vs Fc^+^/Fc, CH_3_CN).
**2.**  ***[Ni(L1)(acac)]NO_3_:(acetylacetonate)(2,2′-(ethane-1,2-diylbis(oxy))bis(N-(4-methoxybenzyl)ethan-1-amine)nickel(II) nitrate*, *(2a).*** Yield: 79%, m.p. 135–138 °C. Elemental analysis calculated for [Ni(C_22_H_32_N_2_O_4_)(C_5_H_8_O_2_)]NO_3_·(CH_3_CN)·(H_2_O)_3_ (704.41 g/mol): C, 49.44; H, 7.01; N, 7.95. Found: C, 49.82; H, 7.23; N, 8.30. IR (ATR, ν/cm^−1^): ʋ(R-NH-R) 3648, ʋ(NH_flex_) 1515, ʋ(C-H_ar_) 3016, 822, ʋ(CH_2_, CH_3_) 2900, 2832, ʋ(C=C) 1464, ʋ(0N0_2_) 1386, ʋ(C-O-C) 1138, ʋ(C-O-C_ar_)_as_ 1249, ʋ(C-O-C_ar_)_sym_ 1038, ʋ(C=O) 1612. Λ_M (CH_3_CN) = 100.2 µS cm^−1^. µ*eef* (BM) = 2.6472. MS, *m*/*z* = 546 (5%). Electrochemical data: E_½_^I^= −1.0763V (vs Fc^+^/Fc, CH_3_CN).
**3.**  ***[Ni(L1)(tfac)]NO_3_: (trifluoroacetylacetonate)(2,2′-(ethane-1,2-diylbis(oxy))bis(N-(4-methoxybenzyl)ethan-1-amine)nickel(II) nitrate*, *(3a).*** Yield: 49%, m.p. 110–115 °C. Elemental analysis calculated for [Ni(C_22_H_32_N_2_O_4_)(C_5_H_4_O_2_F_3_)]NO_3_·(CH_3_CN)·(H_2_O)_3_ (757.37 g/mol): C, 45.98; H, 5.98; N, 7.39. Found: C, 46.02; H, 6.26; N, 7.56. IR (ATR, ν/cm^−1^): ʋ(R-NH-R) 3624, ʋ(NH_flex_) 1514, ʋ(C-H_arm_) 3030, 821, ʋ(CH_2_, CH_3_) 2900, 2832, ʋ(C=C) 1463, ʋ(0N0_2_) 1389, ʋ(C-O-C) 1137, ʋ(C-O-C_arm_)_as_ 1248, ʋ(C-O-C_arm_)_sym_ 1037, ʋ(C=O) 1653, ʋ(C-F) 1296. Λ_M (CH_3_CN) = 148.3 µS cm^−1^. µ*eef* (BM) = 2.4916. MS, *m*/*z* = 596 (15%). Electrochemical data: E_½_^I^ = −1.0541 V(vs Fc^+^/Fc, CH_3_CN).
**4.**  ***[Ni(L1)(hfac)]NO_3_: (hexafluoroacetylacetonate)(2,2′-(ethane-1,2-diylbis(oxy))bis(N-(4-methoxybenzyl)ethan-1-amine)nickel(II) nitrate, (4a).*** Yield: 47%, m.p. 128–132 °C. Elemental analysis calculated for [Ni(C_22_H_32_N_2_O_4_)(C_5_HO_2_F_6_)]NO_3_·(CH_3_CN)·(H_2_O)_2_ (793.33 g/mol): C, 43.90; H, 5.08; N, 7.06. Found: C, 44.35; H, 5.25; N, 7.07. IR (ATR, ν/cm^−1^): ʋ(R-NH-R) 3627, ʋ(NH_flex_) 1515, ʋ(C-H_arm_) 3016, 821, ʋ(CH_2_, CH_3_) 2900, 2831, ʋ(C=C) 1463, ʋ(0N0_2_) 1386, ʋ(C-O-C) 1138, ʋ(C-O-C_arm_)_as_ 1248, ʋ(C-O-C_arm_)_sym_ 1038, ʋ(C=O) 1652, ʋ(C-F) 1297. Λ_M (CH_3_CN) = 115.8 µS cm^−1^. µ*eef* (BM) = 3.0852. MS, *m*/*z* = 653 (65%). Electrochemical data: E_½_^I^ = −1.0413 V(vs Fc^+^/Fc, CH_3_CN).
**5.**  ***[Ni(L2)(NO_3_)_2_]:(Nitrate)(2,2′-etano-1,2-)-3,6-dioxaoctano-1,8-dimina)nickel(II), (1b)*.** Yield: 60%, m.p. 104–106 °C. Elemental analysis calculated for [Ni(C_20_H_28_N_2_O_2_)(NO_3_)_2_](H_2_O)_3_ (565.19 g/mol): C, 42.5; H, 6.06; N, 9.91. Found: C, 42.7; H, 6.16; N, 10.06. IR (ATR, ν/cm^−1^): ʋ(R-NH-R) 3464, ʋ(NH_flex_) 1579, ʋ(C-H_arm_) 3035, 822, ʋ(CH_2_, CH_3_) 2899, 2833, ʋ(C=C) 1427, ʋ(0N0_2_) 1376, 1296, ʋ(C-O-C) 1138. Λ_M (CH_3_CN) = 88.7 µS cm^−1^. µ*eef* (BM) = 3.3499. MS, *m*/*z* = 510 (5%). Electrochemical data: E_½_^I^ = −1.0299 V(vs Fc^+^/Fc, CH_3_CN).
**6.**  ***[Ni(L2)(acac)]NO_3_: (acetylacetonate)(2,2′-(ethane-1,2-diylbis(oxy))bis(N-benzylethan-1-amine)nickel(II) nitrate, (2b)*.** Yield: 62%, m.p. 132–134 °C. Elemental analysis calculated for [Ni(C_20_H_28_N_2_O_2_)(C_5_H_8_O_2_)]NO_3_·(CH_3_CN)·(H_2_O)_3_ (642.34 g/mol): C, 50.48; H, 6.74; N, 8.72. Found: C, 50.63; H, 6.89; N, 9.02. IR (ATR, ν/cm^−1^): ʋ(R-NH-R) 3441, ʋ(NH_flex_) 1596, ʋ(C-H_arm_) 3036, 824, ʋ(CH_2_, CH_3_) 2899, 2856, ʋ(C=C) 1465, ʋ(0N0_2_) 1380, ʋ(C-O-C) 1141, ʋ(C=O) 1676. Λ_M (CH_3_CN) = 109.5 µS cm^−1^. µ*eef* (BM) = 2.4508. MS, *m*/*z* = 546 (15%). Electrochemical data: E_½_^I^ = −1.0348 V(vs Fc^+^/Fc, CH_3_CN).
**7.**  ***[Ni(L2)(tfac)]NO_3_: (trifluoroacetylacetonate)(2,2′-(ethane-1,2-diylbis(oxy))bis(N-benzylethan-1-amine)nickel(II) nitrate, (3b)*.** Yield: 23%, m.p. 90–94 °C. Elemental analysis calculated for [Ni(C_20_H_28_N_2_O_2_)(C_5_H_4_O_2_F_3_)]NO_3_·(CH_3_CN)·(H_2_O)_2_ (679.3 g/mol): C, 47.73; H, 5.78; N, 8.24. Found: C, 48.13; H, 6.05; N, 8.31. IR (ATR, ν/cm^−1^): ʋ(R-NH-R) 3397, ʋ(NH_flex_) 1603, ʋ(C-H_arm_) 3038, 825, ʋ(CH_2_, CH_3_) 2898, 2826, ʋ(C=C) 1450, ʋ(0N0_2_) 1378, ʋ(C-O-C) 1138, ʋ(C=O) 1750, ʋ(C-F) 1309. Λ (CH_3_CN) = 145.0 µS. µ*eef* (BM) = 2.7409. MS, *m*/*z* = 538 (65%). Electrochemical data: E_½_^I^ = −1.0366 V(vs Fc^+^/Fc, CH_3_CN).
**8.**  ***[Ni(L2)(hfac)]NO_3_: (hexafluoroacetylacetonate)(2,2′-(ethane-1,2-diylbis(oxy))bis(N-benzylethan-1-amine)nickel(II) nitrate, (4b)*.** Yield: 23%, m.p. 119–123 °C. Elemental analysis calculated for [Ni(C_20_H_28_N_2_O_2_)(C_5_HO_2_F_6_)]NO_3_·(CH_3_CN)·(H_2_O)_2_ (733.28 g/mol): C, 44.22; H, 4.94; N, 7.64. Found: C, 44.87; H, 6.15; N, 7.9 IR (ATR, ν/cm^−1^): ʋ(R-NH-R) 3386, ʋ(NH_flex_) 1597, ʋ(C-H_arm_) 3035, 823, ʋ(CH_2_, CH_3_) 2898, 2855, ʋ(C=C) 1471, ʋ(0N0_2_) 1380, ʋ(C-O-C) 1137, ʋ(C=O) 1744, ʋ(C-F) 1309. Λ_M (CH_3_CN) = 130.9 µS cm^−1^. µ*eef* (BM) = 1.8133. MS, *m*/*z* = 593 (25%). Electrochemical data: E_½_^I^ = −1.0379 V(vs Fc^+^/Fc, CH_3_CN).

### 3.4. X-Ray Crystallography

A suitable single crystal of ligand 2,2′-(ethane-1,2-diylbis(oxy))bis(N-(4-methoxybenzyl)ethane-1-aminium), **L1** was mounted on a glass fiber and crystallographic data were collected at 130 K with an Oxford Diffraction Gemini diffractometer with monochromator of graphite using λ_MoKα_ = 0.71073 Å, equipped with a CCD-atlas area detector. CrysAlisPro and CrysAlis RED software packages were used for data collection and integration [68]. The double pass method of scanning was used to exclude any noise. The collected frames were integrated by using an orientation matrix determined from the narrow frame scans. Final cell constants were determined by global refinement; data were collected and corrected for absorbance using analytical numerical absorption correction with a multifaceted crystal model based on expressions upon the Laue symmetry with equivalent reflections [69]. The solution and refinement of the structure were carried out with the SHELXS-2018/3 [70] and SHELXL-2018/3 [71] packages. WinGX v2023 [72] software was used to prepare material for publication. Full-matrix least-squares refinement was carried out by minimizing (*Fo*^2^–*Fc*^2^)^2^. All non-hydrogen atoms were refined anisotropically. H atoms of amine (N-H) groups were located in a difference map and refined isotropically with Uiso(H) 1.2 Ueq for N-H. H atoms attached to C atoms were placed in geometrically idealized positions and refined as riding on their parent atoms, with C–H = 0.95–0.99 Å with *U_iso_*(H) = 1.2*U_eq_*(C) for aromatic and methylene groups and *U_iso_*(H) = 1.5*U_eq_*(C) for methyl groups. The C1 C2 C3 C4 C5 C6 O1 C11 and C1P C2P C3P C4P C5P C6P O1P C11P are disordered over two sites with occupancies of 0.62:0.38. Table 6 shows the crystallographic data summary.

The crystallographic data has been deposited with the Cambridge Crystallographic Data Centre as supplementary publication no. CCDC 2545367. Copies of the data can be obtained free of charge on application to CCDC, 12 Union Road, Cambridge, CB2 1EZ, UK (fax: (+44) 1223-336-033, e-mail: deposit@ccdc.cam.ac.uk).

### 3.5. Computational Details

DFT calculations were performed using Gaussian 16 [73]. Initial structures were drawn in GaussView^®^ considering the physicochemical characterization as distorted octahedra. All Ni(II) complexes were modeled as cationic triplet species, using the UB3LYP functional [74]. The LANL2DZ/effective core potential basis set was used for Ni [75], while 6-311 + G(d,p) was used for all non-metal atoms [76]. Geometry optimizations and harmonic frequency calculations were performed on acetonitrile using the SMD solvation model [77]. The absence of imaginary frequencies confirmed that the optimized structures were true minima.

Electronic spectra were calculated from the optimized structures using TD-DFT at the same level of theory, including the first 60 vertical excited states [78]. Simulated UV-Vis spectra were generated by gaussian convolution of discrete transitions with a width-to-half-height of 0.30 eV using GaussView^®^ 6.0 (Semichem Inc., Shawnee Mission, KS, USA).

### 3.6. Electrochemical Studies

All electrochemical measurements were performed in anhydrous acetonitrile (HPLC grade) containing 0.1 M tetrabutylammonium hexafluorophosphate (TBAPF_6_, Sigma-Aldrich, 99.99%) as the supporting electrolyte. Cyclic voltammograms were recorded using a PAR Model 273 potentiostat/galvanostat (Princeton Applied Research, Oak Ridge, TN, USA) equipped with a conventional three-electrode cell consisting of a platinum working electrode, a platinum wire auxiliary electrode, and a silver wire pseudoreference electrode. Prior to each measurement, the platinum working electrode was mechanically polished with diamond paste, and the solutions were purged with nitrogen for 10 min to remove dissolved oxygen. Ohmic drop (iR) compensation was applied using the positive feedback method. Ferrocene (99.9%, Sigma-Aldrich, St. Louis, MO, USA) was added as an internal reference after each experiment, and all potentials are reported versus the ferrocenium/ferrocene (Fc^+^/Fc) redox couple, following IUPAC recommendations for non-aqueous electrochemical measurements [42].

### 3.7. Antiproliferative Activity

The adherent HeLa cell line (CCL-2, American Type Culture Collection, Rockville, MD, USA) was used. Cells were cultured in RPMI 1640 medium containing 2.5 mM L-glutamine, 25 mM HEPES, supplemented with 10% fetal bovine serum (FBS, Biowest, Nuaillé, France), 1% antibiotic and antifungal (Anti-Anti; Biowest, Nuaillé, France), and 1% non-essential amino acids (NEA, Biowest, Nuaillé, France), in sterile 75 cm^2^ Corning culture flasks and incubated at 37 °C in a humidified atmosphere containing 5% CO_2_ using a CO_2_ cell incubator (MMM Group, Planegg, Germany). Cell growth was determined according to the sulforhodamine B assay [47].

The treatments were administered in quadruplicate to each plate. The compounds were dissolved in sterile water plus 10% acetonitrile and the concentrations evaluated for the mixed compounds were 200, 150, 100, 50, and 10 μM to determine the IC_50_. The cells were exposed to the coordination compounds for 24 h, fixed with 10% trichloroacetic acid, and then fixed with sulforhodamine B (4% *w*/*v* in 1% *v*/*v* acetic acid). The sulforhodamine was redissolved using Trizma base (pH 10) and quantified using a microplate reader at 570 nm. Using the absorbance data obtained, a dose–response curve was constructed with the concentration values in micromolar vs. percentage of cell inhibition, and the IC_50_ was determined.

### 3.8. Mechanisms of Action Assay

The compound **4a** was dissolved in sterile water plus 10% acetonitrile. For the different determinations, the mean inhibitory concentration of 56.00 µM, which had been previously determined, was used. Subsequently, 1 × 10^6^ cells were treated for 30 min, 1, 2, 3, 4, and 6 h with the IC_50_; the negative control consisted of untreated cells.

#### 3.8.1. Caspase-3 Study

To determine the effect of the compound, we detected the presence of caspase-3 using the Dot Blot immunoassay with a time range of 30 min to 6 h. Starting with 1 × 10^6^ cells, protein extraction was performed using Tris-HCl buffer, pH 7.0, with protease inhibitors. One microliter of each sample at a concentration of 15 µg/mL was loaded in triplicate onto nitrocellulose membranes labeled within 1 cm circles. Two 10 min washes were performed with TBS. Subsequently, the membrane was blocked with 5% Svelty milk for 15 min. Three 10 min washes were then performed with TTBS. The membranes were incubated with the primary antibody caspase-3 (sc-7272, Santa Cruz Biotechnology, Dallas, TX, USA) and β-actin (sc-130065, Santa Cruz Biotechnology, Carpinteria, CA, USA) as a loading control, at a 1:1000 dilution for 2 h. After this time, the membranes were washed three times with TTBS for 10 min each. They were then incubated with the secondary antibody goat anti-mouse (sc-2005, Santa Cruz Biotechnology, Dallas, TX, USA) at a 1:5000 dilution for two hours. After this time, they were washed three times with TTBS for 10 min each and twice with TBS for 5 min each. Finally, the proteins were detected by chemiluminescence for which they were revealed with ECL for 0.3 min, and observed in a Bio-Rad Chemidoc Image System reader.

#### 3.8.2. Oxidative Stress Assessment

2 × 10^4^ cells were cultured in 96-well plates and treated with the IC_50_ of **4a** for 30 min. The cells were first washed with 1X phosphate-buffered saline (PBS). The superoxide (O_2_^•−^) concentration was determined using MitoSox™ reagent (ThermoFisher Scientific, Waltham, MA, USA), a hydroethidine analog. The cells were incubated with 250 µL of DMSO diluted to 5 μM stock solution for 10 min in the dark at 37 °C. As a positive control, the cells were irradiated with UV light for 3 h. Oxidation products were detected by fluorescence using a Varioskan plate reader (Thermo Fisher Scientific, Waltham, MA, USA; Ex/Em 510/580 nm).

#### 3.8.3. Statistical Analysis

Data are presented as mean ± standard deviation (SD). All methodologies were performed in triplicate, and differences between groups were assessed using analysis of variance (ANOVA). Data were analyzed using GraphPad Prism Version 8.0.0 statistical software (La Jolla, CA, USA). A *p*-value less than 0.05 was considered statistically significant.

## 4. Conclusions

Two N_2_O_2_-type ligands (**L1** and **L2**) were successfully synthesized, optimized, and characterized through environmentally friendly routes within a green chemistry framework. Their coordination with Ni(II) afforded a series of octahedral mixed-ligand complexes incorporating nitrate or β-diketonate secondary ligands, including fluorinated derivatives. All complexes exhibit a d^8^ electronic configuration, and the mixed species (**2a**–**4a** and **2b**–**4b**) behave as 1:1 electrolyte in solution.

Electrochemical studies showed that the Ni(II)/Ni(I) redox behavior is strongly influenced by the coordination environment and can be effectively tuned through ligand modification. The quasi-reversible redox response and the systematic shifts in cathodic peak potentials (Epc) demonstrate that both the tetradentate Schiff base and the coordinated β-diketonate ligands modulate the electron density at the nickel center.

Variations in electronic properties were associated with differences in biological activity. The highest antiproliferative activity in HeLa cells was observed for the **L1**-based complex containing a perfluorinated β-diketonate (**4a**), suggesting that the combination of a methoxy-substituted ligand framework and an electron-withdrawing secondary ligand favors biological performance relative to the unsubstituted analogues. Mechanistic studies support apoptosis induction through a redox-associated pathway, as evidenced by early caspase-3 activation and apoptosis-related morphological changes, which may be associated with the generation of reactive oxygen species (ROS).

Overall, these findings demonstrate that controlled modification of the ligand environment represents an effective strategy to tune the electronic, electrochemical, and biological properties of Ni(II) mixed-ligand complexes.

## Figures and Tables

**Scheme 1:**
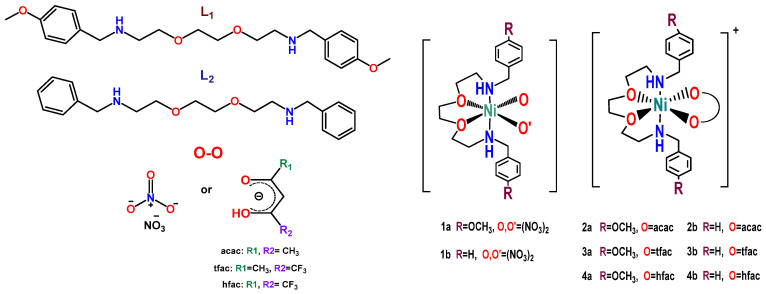
Structures of hydrogenated tridentate ligands derived from Schiff bases = (N_2_O_2_) (primary ligands) and β-diketones = acac with fluorinated substituents (secondary ligands) and the general structure of the mixed complexes of [Ni(N_2_O_2_)(O-O)]NO_3_.

**Scheme 2:**
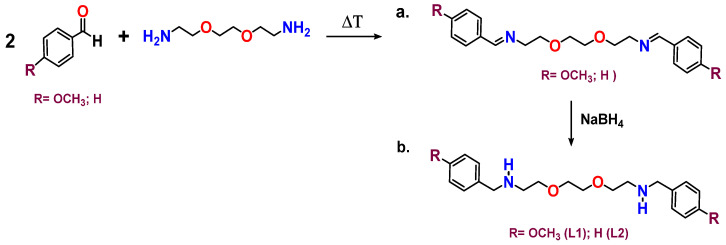
Synthetic route for the preparation of the hydrogenated Schiff base ligands **L1** and **L2**. (**a**) Formation of the Schiff base; (**b**) Hydrogenation of the Schiff base.

**Figure 1:**
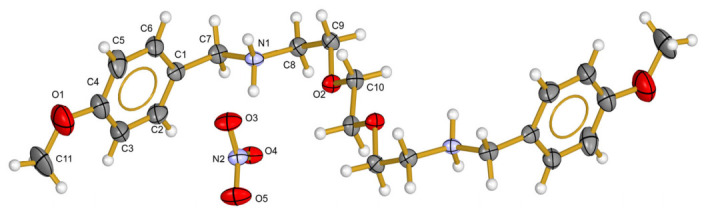
ORTEP drawing of the molecule discrete for Schiff base ligand 2,2′-[ethane-1,2-diylbis(oxy)]bis{N-[(4-methoxyphenyl)methyl]ethan-1-aminium} dinitrate (**L1**), with the thermal ellipsoids drawn at the 60% of probability.

**Figure 2:**
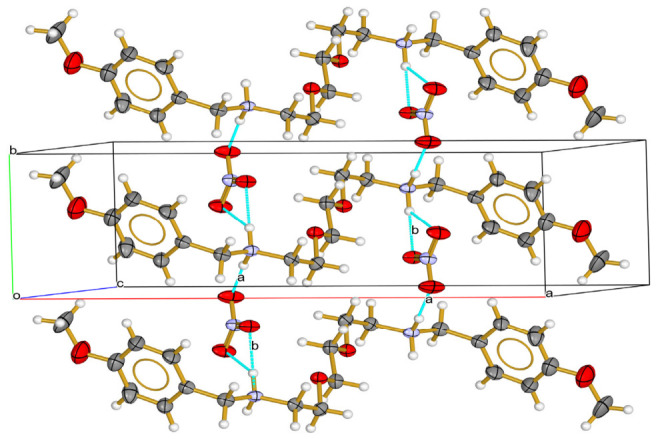
Crystal array of the compound **L1**, viewed along the plane *a-b* emphasizing the N-H…O intermolecular interaction.

**Scheme 3:**
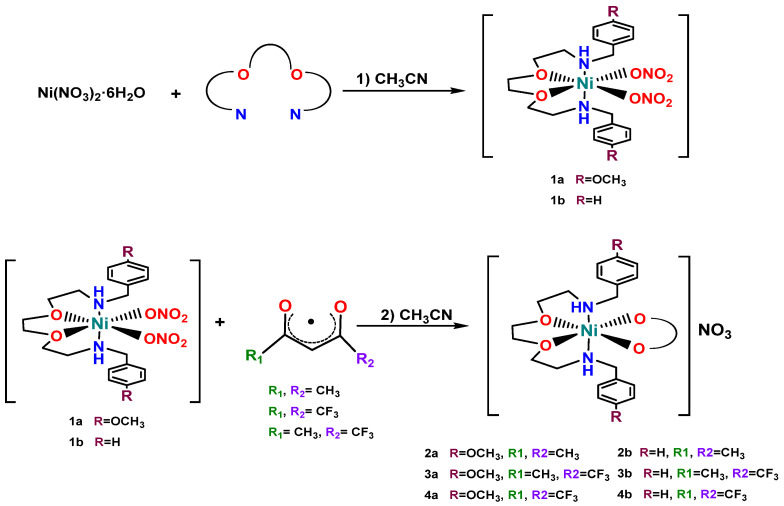
General scheme of the synthesis of mixed octahedral coordination complexes of Ni(II) with N_2_O_2_ ligands and diketones.

**Figure 3:**
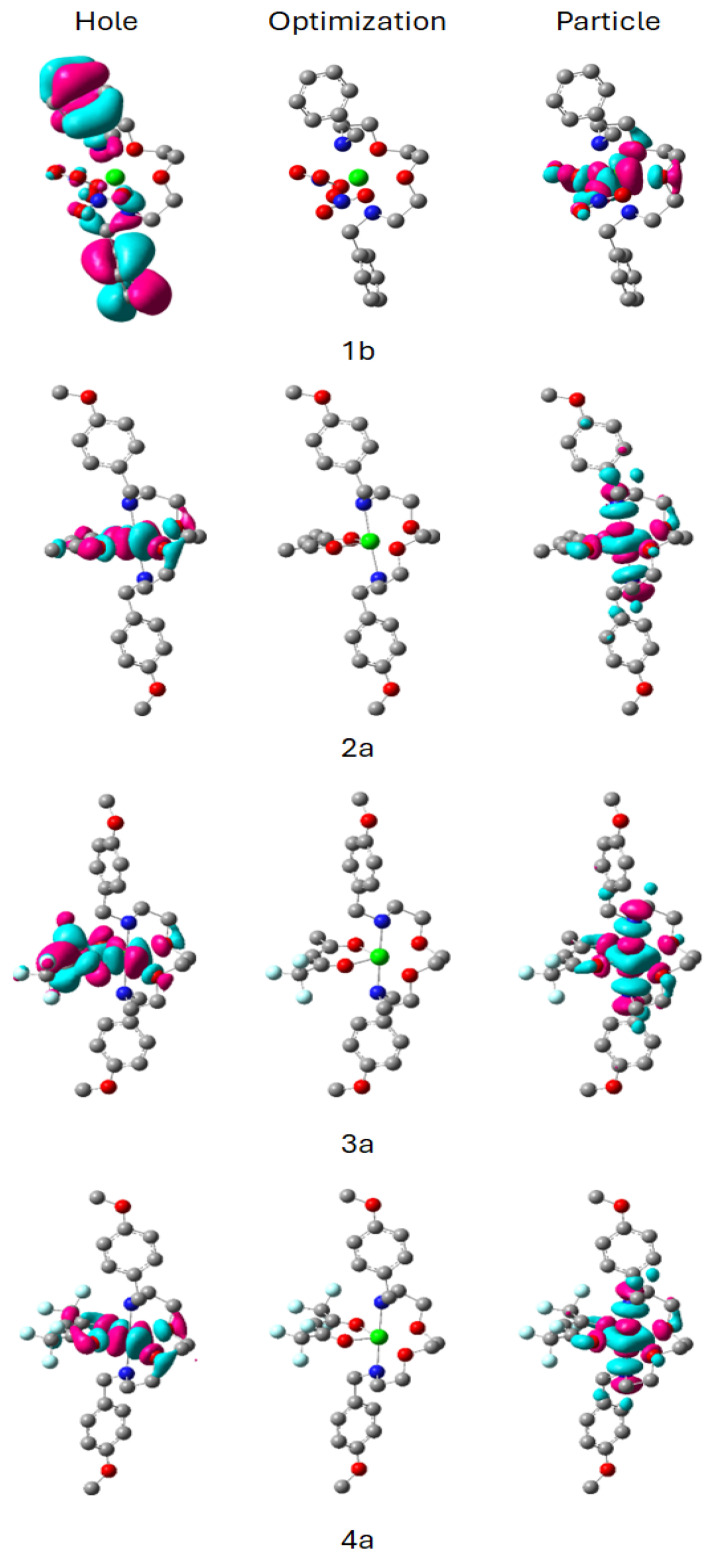
NTOs for **1b** and **2a**–**4a** complexes. In the center of the image the optimization is shown as guidance. Hydrogen atoms were omitted for clarity.

**Figure 4:**
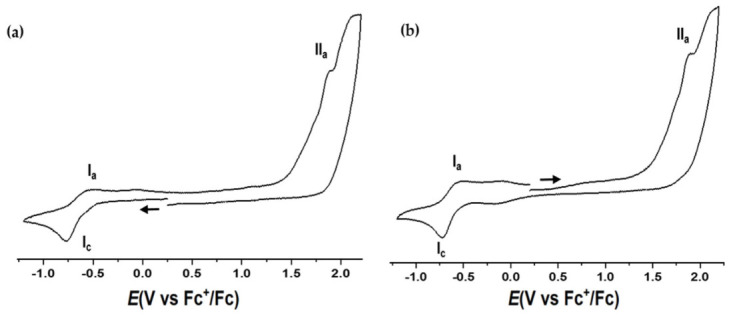
Cyclic voltammogram of **1a** [NiL1]NO_3_ (2.0 mM) recorded in CH_3_CN containing 0.1 M TBAPF_6_ using a Pt working electrode at a scan rate of 300 mV s^−1^. The potential scan was initiated at the open-circuit potential and performed toward the negative (**a**) and positive (**b**) potential directions. The arrows indicate the direction of the potential scan. Potentials are referenced to the Fc^+^/Fc redox couple.

**Figure 5:**
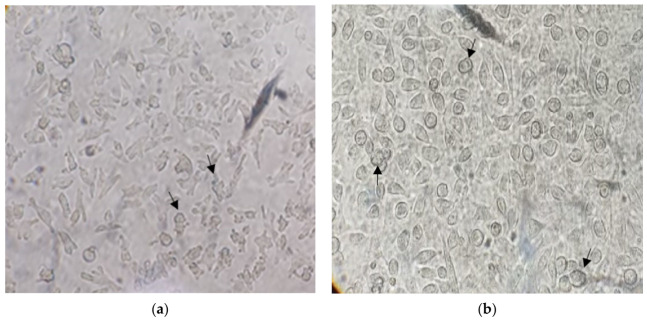
The arrows show HeLa cells treated with the IC_50_ of **4a** and viewed at 20× magnification at two specific times: (**a**) 3 h post-treatment showing blebbing, an early form of apoptotic bodies, and (**b**) 4 h post-treatment, where more rounded cells are observed, consistent with apoptosis at longer times.

**Figure 6:**
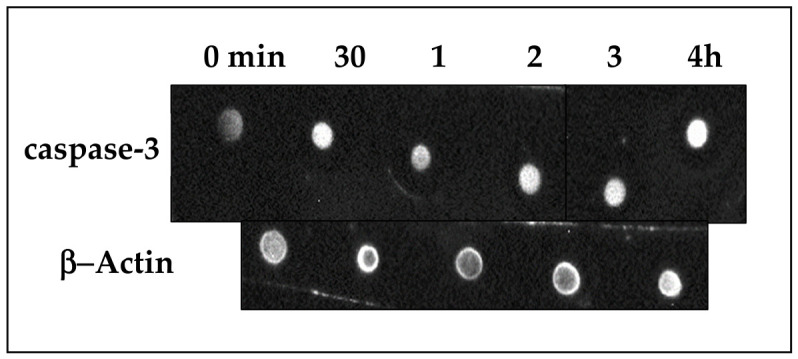
Dot-Blot showing the presence of caspase-3 from 30 min of treatment, up to 4h. It was compared with the presence of β-actin as a load control.

**Figure 7:**
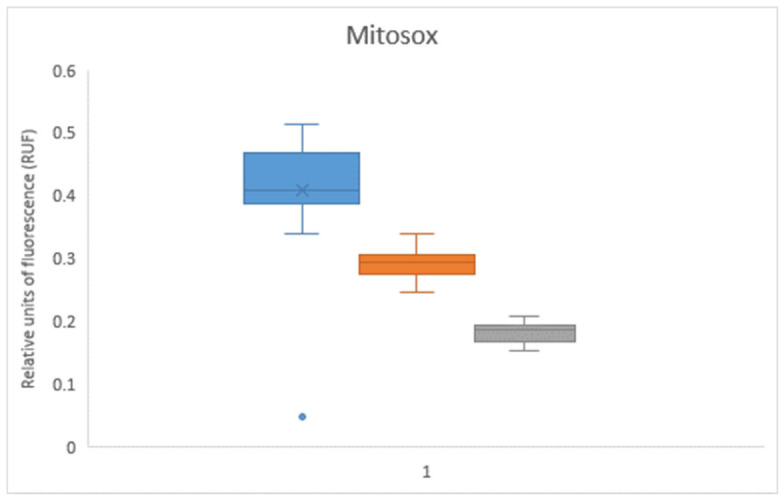
The pro-oxidant state of the compound under study was analyzed by measuring superoxide expression after 30 min of exposure, compared with cells irradiated with UV light for three hours (positive control, blue), untreated cells (negative control, orange) and cells treated with compound **4a** (IC_50_, gray). The differences were statistically significant (*p* < 0.05).

**Table 1:** Selected bond lengths [Å] and angles [°] for **L1**.

Bond	(Å)	Angle	(°)
C(1)-C(2)	1.394(7)	C(10)-O(2)-C(9)	112.51(12)
C(1)-C(7)	1.522(6)	C(2)-C(1)-C(7)	121.2(5)
N(1)-C(7)	1.499(2)	C(4)-O(1)-C(11)	119.1(5)
N(1)-C(8)	1.490(2)	C(6)-C(1)-C(2)	117.0(5)
N(2)-O(3)	1.2304(19)	C(6)-C(1)-C(7)	121.9(5)
N(2)-O(4)	1.2564(18)	C(8)-N(1)-C(7)	113.25(13)
O(1)-C(11)	1.416(6)	N(1)-C(7)-C(1)	107.9(5)
O(1)-C(4)	1.379(5)	N(1)-C(8)-C(9)	109.77(13)
O(2)-C(10)	1.4204(19)	O(2)-C(9)-C(8)	107.57(13)
O(2)-C(9)	1.421(2)	O(3)-N(2)-O(4)	120.69(14)

**Table 2:** Main ATR-IR absorption bands (cm^−1^).

Code	ʋ(N-H)*_flex_* (cm^−1^)	ʋ C-O-C) (cm^−1^)	ʋ(C-O-C_ar_)*_asym_* (cm^−1^)	ʋ(C-O-C_ar_)*_sym_* (cm^−1^)	ʋ(N-O) (cm^−1^)	ʋ(C=O) (cm^−1^)	ʋ(C-F) (cm^−1^)
**1a**	3642 1514	1137	1248	1032	1300	-	-
**2a**	3648 1515	1138	1249	1038	1386	1694	-
**3a**	3624 1514	1137	1248	1037	1389	1654	1296
**4a**	3627 1515	1138	1248	1038	1386	1652	1297
**1b**	3464 1579	1138	-	-	1303	-	-
**2b**	3441 1596	1140	-	-	1380	1687	-
**3b**	3397 1603	1138	-	-	1378	1750	1309
**4b**	3386 1597	1137	-	-	1380	1744	1309

**Table 3:** Electronic absorption data of the Ni(II) complexes recorded in CH_3_CN. Wavelengths (λ) are given in nm; values in parentheses correspond to the molar extinction coefficients, ε (L mol^−1^ cm^−1^). Diffuse reflectance spectroscopy (DRS) bands are reported in cm^−1^.

Code	λ (π-π*) N_2_O_2_ nm	λ (π-π*) N_2_O_2_ nm	λ (π-π*) β Diketone nm	λ (CT) -ONO_2_ nm	^3^A_2_g → ^3^T_2_g(F) cm^−1^	^3^A_2_g → ^3^T_1_g(F) cm^−1^	^3^A_2_g → ^3^T_1_g(P) cm^−1^
**1a**	225 (8241)	274 (6850)	-	383 (161)	8187	14,856	25,643
**2a**	225 (8035)	273 (6825)	316 (161)	-	8208	14,843	25,670
**3a**	226 (7902)	273 (6798)	310 (272)	-	8386	14,884	25,773
**4a**	226 (8593)	274 (6308)	308 (317)	-	8297	14,809	25,629
**1b**	207 (9857)	257 (6167)	-	386 (167)	8379	14,836	25,390
**2b**	206 (9074)	257 (6174)	313 (159)	-	8427	14,959	25,417
**3b**	205 (9970)	256 (6198)	308 (167)	-	8502	14,897	25,725
**4b**	205 (9703)	257 (6089)	306 (125)	-	8441	14,678	25,595

**Table 4:** Electrochemical data for nickel(II) complexes (2.0 mM) recorded in CH_3_CN at a scan rate of 300 mV s^−1^. Potentials are referenced to the Fc^+^/Fc redox couple.

Compound	*E_p_*_,_*_a_* (V vs. Fc^+^/Fc)	*E_p_*_,_*_c_* (V vs. Fc^+^/Fc)	Δ*E_p_* (mV)	I_pa_/I_pc_
**1a**	−1.013	−1.115	102.0	0.78
**2a**	−1.029	−1.107	78.0	0.65
**3a**	−1.031	−1.116	85.0	0.93
**4a**	−1.036	−1.126	90.0	0.83
**1b**	−0.993	−1.067	74.0	1.01
**2b**	−1.003	−1.079	76.0	0.84
**3b**	−1.017	−1.106	89.0	0.86
**4b**	−1.018	−1.091	73.0	0.78

**Table 5:** Percentage inhibition and IC_50_ values for HeLa cell proliferation treated with mixed nickel compounds.

Compounds	IC_50_ (μM)	Compounds	IC_50_ (μM)
**1a**	139.7 ± 26.2	**1b**	>200
**2a**	79.6 ± 13.3	**2b**	>200
**3a**	71.9 ± 7.9	**3b**	>200
**4a**	56.0 ± 8.2	**4b**	>200
**L1**	>200	**L2**	>200

**Table 6:** Crystal data and structure refinement for as 2,2′-(ethane-1,2-diylbis(oxy))bis(N-(4-methoxybenzyl)ethane-1-aminium), **L1**.

Identification Code	L1
Empirical formula	C_11_ H_17_ N_2_ O_5_
Formula weight	257.26
Temperature	130(2) K
Wavelength	0.71073 Å
Crystal system	Monoclinic
Space group	P 21/c
Unit cell dimensions	a = 20.0912(18) Å
	b = 5.7852(5) Å
	c = 11.2051(9) Å
	β = 95.621(8)°.
Volume	1296.12(19) Å^3^
Z	4
Density (calculated)	1.318 Mg/m^3^
Absorption coefficient	0.105 mm^−1^
F(000)	548
Crystal size	0.560 × 0.480 × 0.130 mm^3^
Theta range for data collection	3.654 to 29.421°.
Index ranges	−27 ≤ h ≤ 22, −7 ≤ k ≤ 6, −14 ≤ l ≤ 12
Reflections collected	6453
Independent reflections	3065 [R(int) = 0.0302]
Completeness to theta = 25.242°	99.9%
Refinement method	Full-matrix least-squares on F^2^
Data/restraints/parameters	3065/16/232
Goodness-of-fit on F^2^	1.134
Final R indices [I>2sigma(I)]	R1 = 0.0555, wR2 = 0.1596
R indices (all data)	R1 = 0.0794, wR2 = 0.1820
Largest diff. peak and hole	0.248 and −0.260 e.Å^−3^

## Data Availability

The crystallographic data reported in this study have been deposited with the Cambridge Crystallographic Data Centre (CCDC, Cambridge, UK) under deposition number **CCDC 2545367** and can be obtained free of charge via the CCDC Access Structures service (https://www.ccdc.cam.ac.uk/structures/ accessed on 13 July 2026). The remaining data supporting the findings of this study, including the original spectroscopic, electrochemical, computational, and biological data, are available from the corresponding author upon reasonable request.

## Supplementary Materials

The following supporting information can be downloaded at: https://www.mdpi.com/article/10.3390/ijms27156601/s1.

## Abbreviations

The following abbreviations are used in this manuscript:

DNA	Deoxyribonucleic Acid
IC_50_	Half Maximal Inhibitory Concentration
SRB	Sulforhodamine B
ROS	Reactive Oxygen Species
GSH	Glutathione
GPx	Glutathione peroxidase
SOD	Superoxide Dismutase
RPMI	Roswell Park Memorial Institute (medium)
HEPES	4-(2-hydroxyethyl)-1-piperazineethanesulfonic acid
FT-IR	Fourier Transform Infrared Spectroscopy
ATR-IR	Attenuated Total Reflectance Infrared Spectroscopy
DRS	Diffuse Reflectance Spectroscopy
ε	Molar Extinction Coefficient/Molar Absorptivity
CT	Charge Transfer
iR	Current × Resistance (Ohmic drop)
IUPAC	International Union of Pure and Applied Chemistry
EC	Electrochemical (mechanism) o Electrochemical step followed by Chemical step
Fc^+^/Fc	Ferrocene/Ferrocenium Redox Couple
Epa	Anodic Peak Potential
Epc	Cathodic Peak Potential
FAB^+^	Fast Atom Bombardment (positive mode)
M/Z^+^	Mass-to-Charge Ratio (relative intensity percentage)
Λ	Molar Conductivity
TD-DFT	Time-dependent density functional theory
