# Supplementary material for: Ni(II) Complexes with Mixed Ligands, Reduced N2O2 Schiff Bases and β-Diketones: Redox Modulation, ROS Generation and Antiproliferative Activity in Cancer Cells Associated with Caspase-3
Source: Int J Mol Sci. 2026 Jul 24;27(15):6601. doi: 10.3390/ijms27156601 (PMC13465113; doi:10.3390/ijms27156601)
Supplement: Supplementary file 1 [file ijms-27-06601-s001.zip › ijms-4371333-supplementary.pdf]

International Journal of Molecular Sciences (Article)

Ni(II) complexes with mixed ligands, reduced N_2O_2 Schiff bases and β-diketones: redox modulation, ROS generation and antiproliferative activity in cancer cells associated with caspase-3

Erika Lorena Cedillo-Gutiérrez [1], Adrián Espinoza-Guillén[1], Luis Felipe Hernández-Ayala[1], Esther Reveles-Ayala[4] Marcos Flores-Alamo[2], Luis Antonio Ortiz-Frade[3], Carmen Mejía[4], Lena Ruiz-Azuara*[1].

[1]Facultad de Química, Universidad Nacional Autónoma de México, Av. Universidad 3000, Circuito Exterior s/n, CU, Ciudad de México C.P. 04510, México

[2]Laboratorio de Biomedicina Interdisciplinaria, Centro Interdisciplinario de Biociencias Aplicadas a la Salud, Facultad de Ciencias Naturales, Universidad Autónoma de Querétaro, Querétaro C.P. 76230, México

[3]Edificio H Mario Molina, Circuito Mario de la Cueva, Esquina Circuito de la Investigación Científica, CU, Ciudad de México C.P. 04510, México

[4]Centro de Investigación y Desarrollo Tecnológico en Electroquímica (CIDETEQ), Parque Tecnológico Quéretaro s/n, Sanfandila, Pedro Escobedo, Querétaro C.P. 76703, México

Supplementary material

Ligands N_2O_2

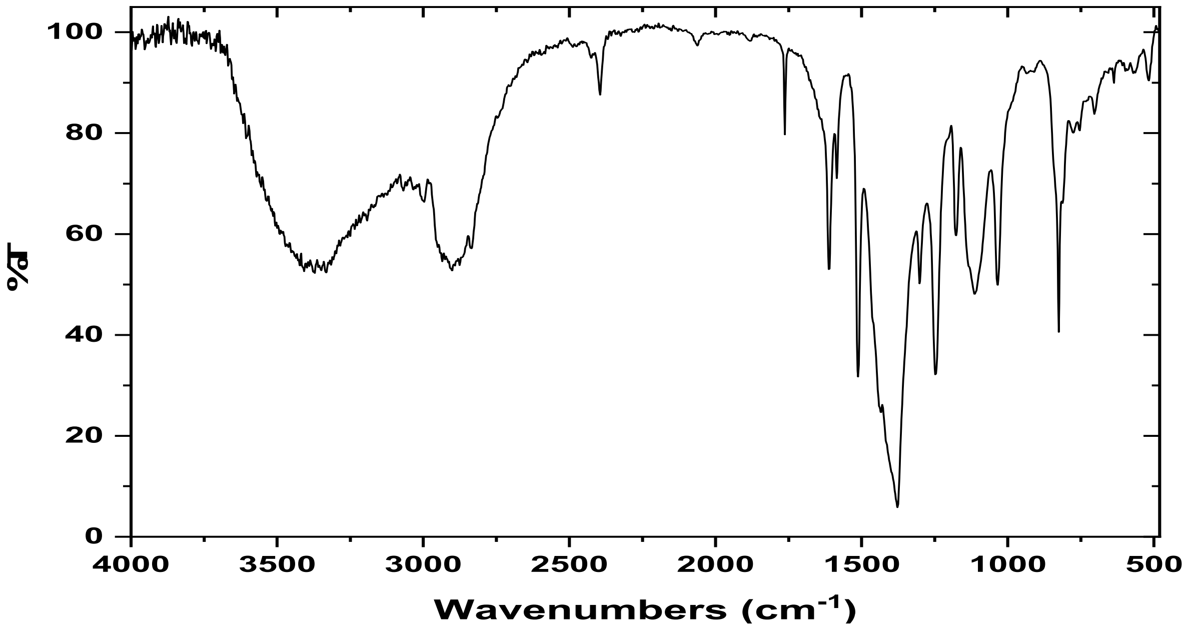

Figure S1. Infrared spectrum (400–4000 cm^{-1}) of the ligand **L1** obtained from as 2,2'-(ethane-1,2-diylbis(oxy))bis(N-(4-methoxybenzyl)ethane-1-amine).

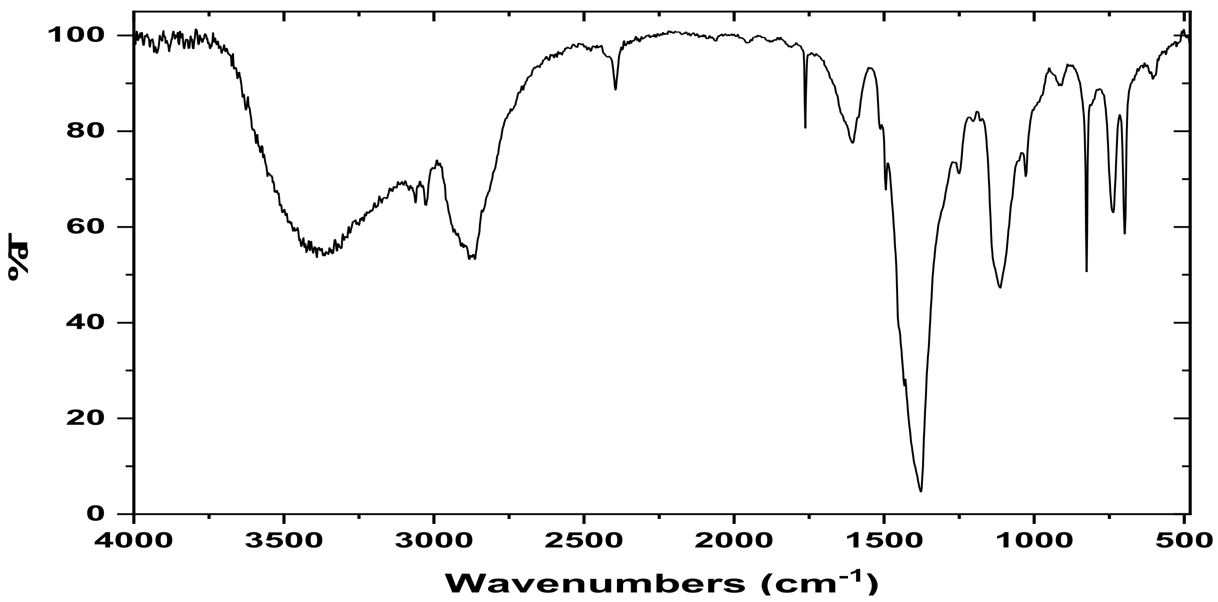

Figure S2. Infrared spectrum (400–4000 cm^{-1}) of the ligand **L2** obtained from 2,2'-(ethane-1,2-diylbis(oxy))bis(N-benzylethan-1-amine).

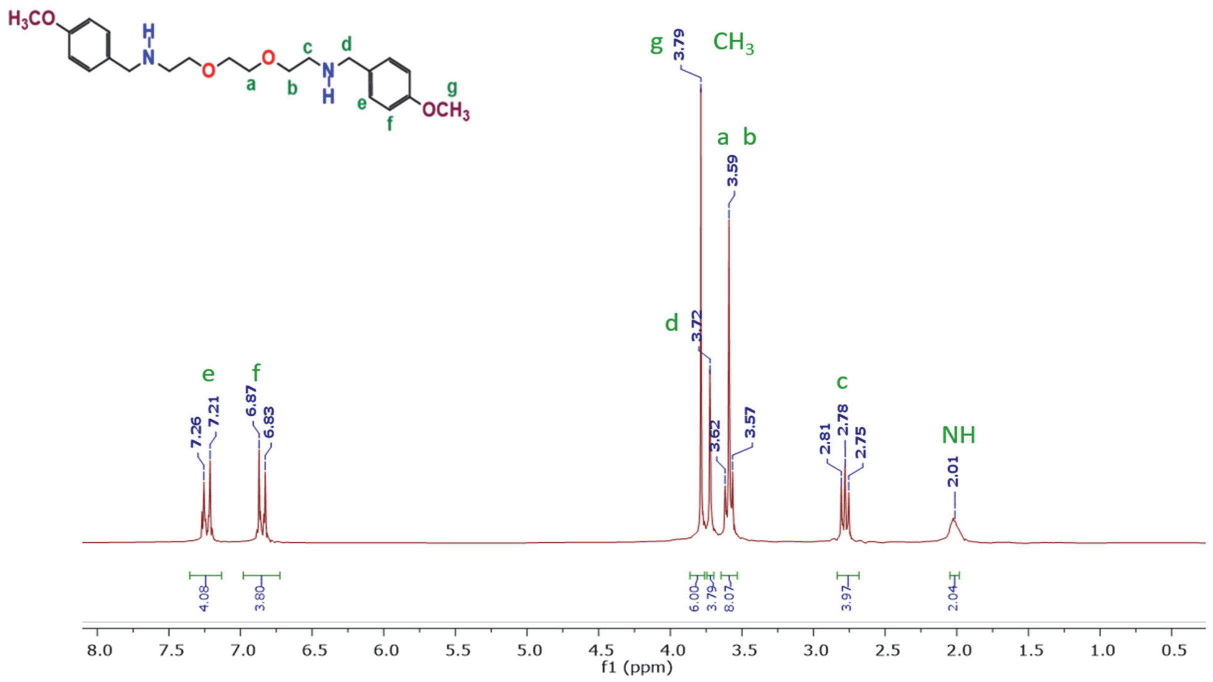

Figure S3. ^{1}H NMR (200 MHz, $CDCl_3$, TMS) Spectrum of ligand 2,2'-(ethane-1,2-diylbis(oxy))bis(N-(4-methoxybenzyl)ethane-1-amine), **L1**

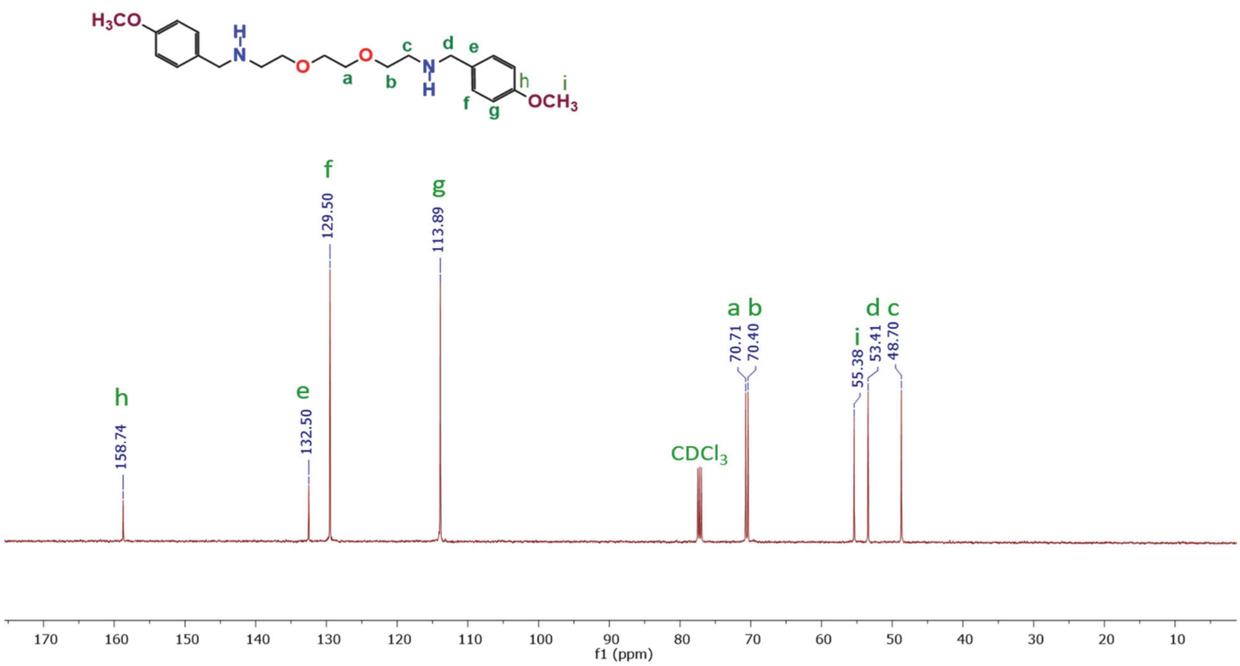

Figure S4. ^{13}C-NMR (50 MHz, $CDCl_3$, TMS) Spectrum of ligand 2,2'-(ethane-1,2-diylbis(oxy))bis(N-(4-methoxybenzyl)ethane-1-amine), **L1**

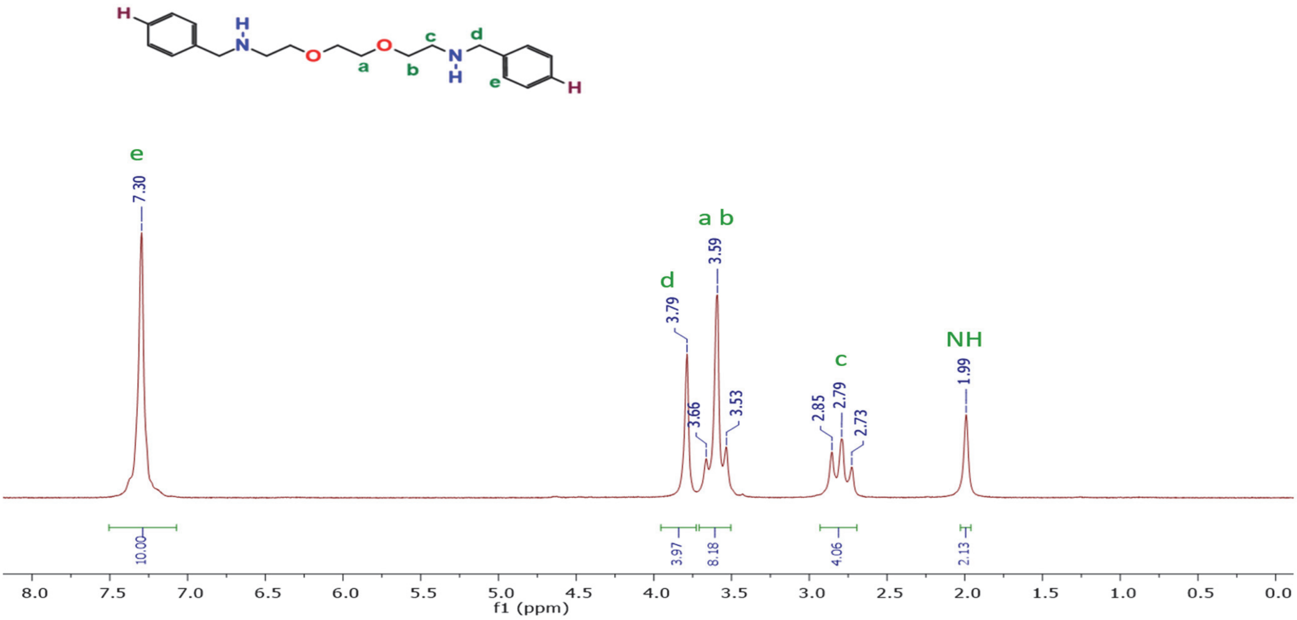

Figure S5. ^{1}H NMR (300 MHz, $CDCl_3$, TMS) Spectrum of ligand 2,2'-(ethane-1,2-diylbis(oxy))bis(N-benzylethan-1-amine), **L2**

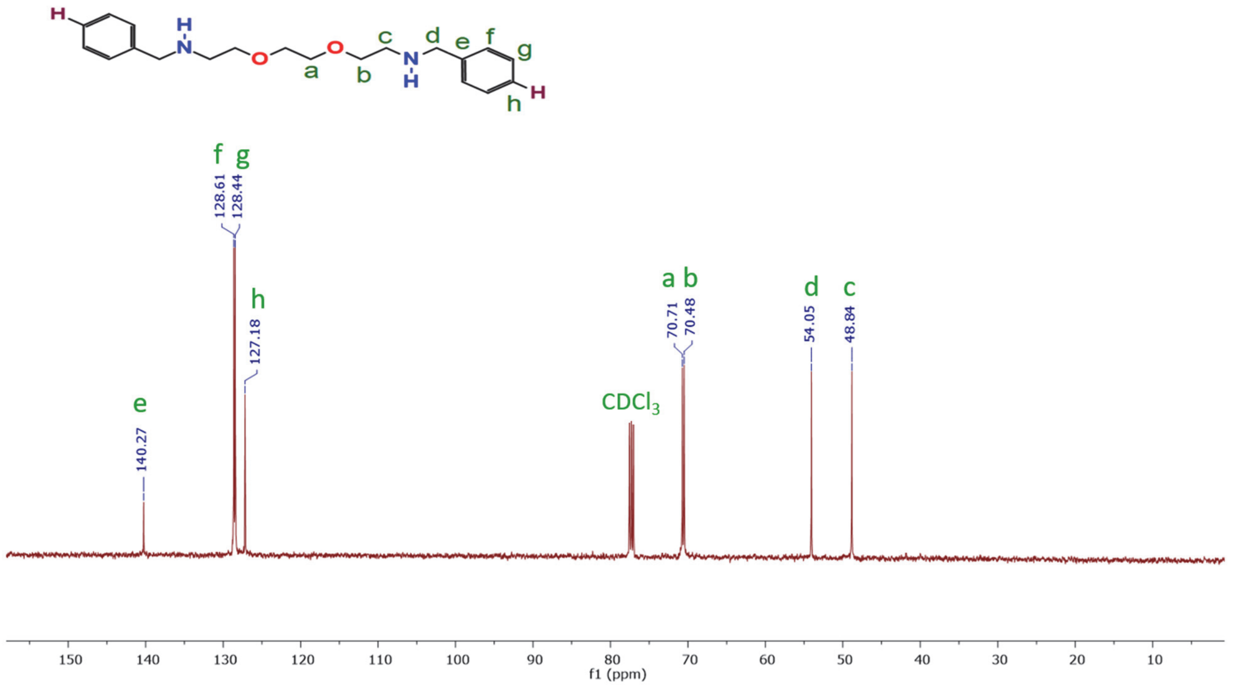

Figure S6. ^{13}C NMR (75 MHz, $CDCl_3$, TMS) Spectrum of ligand 2,2'-(ethane-1,2-diylbis(oxy))bis(N-benzylethan-1-amine), **L2**

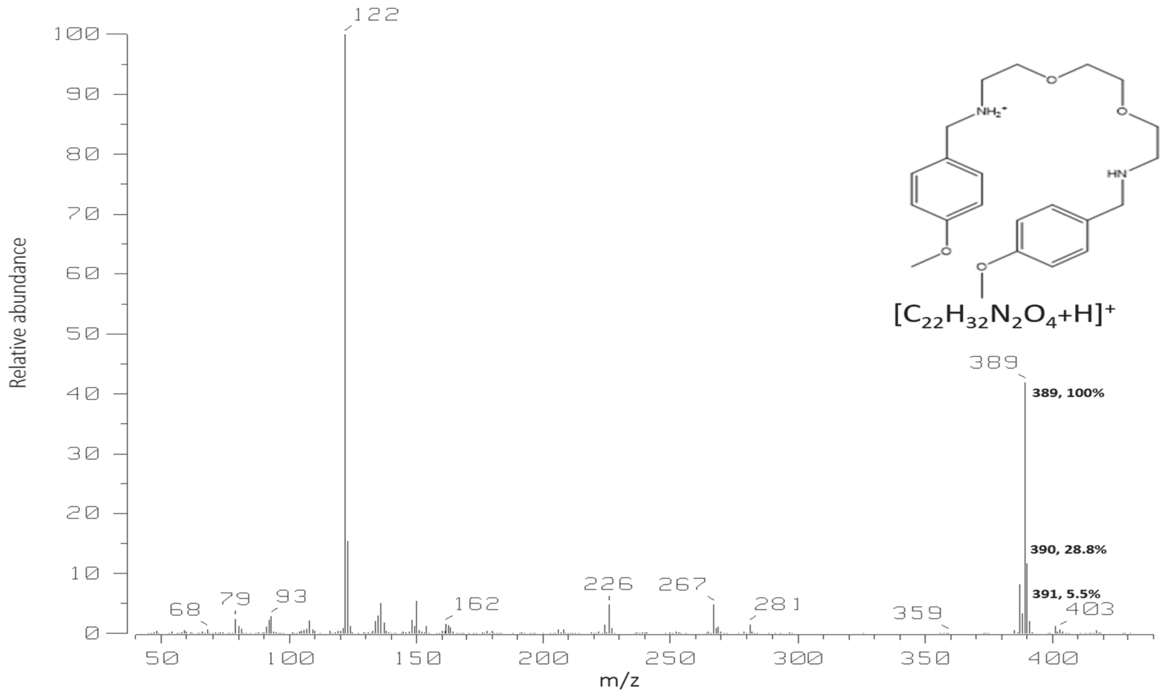

Figure S7. FAB$^+$ mass spectrum of ligand **L1,** [2,2′-(ethane-1,2-diylbis(oxy))bis(N-(4-methoxybenzyl)ethane-1-amine)] recorded in acetonitrile.

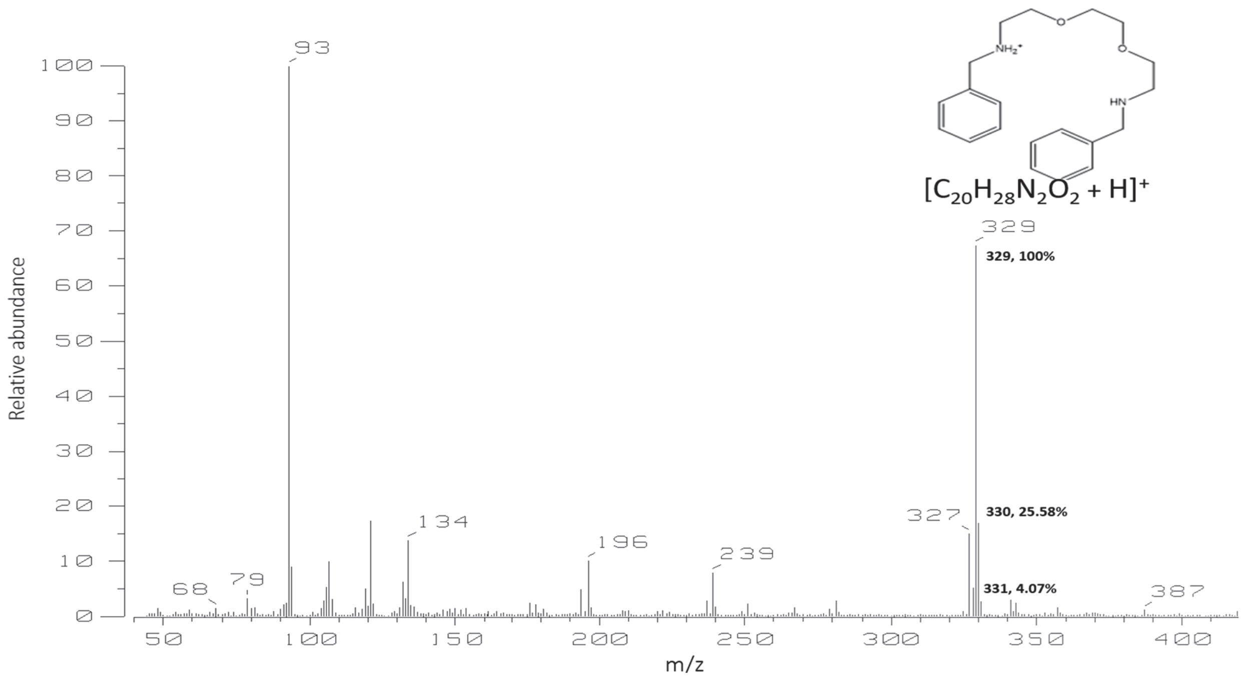

Figure S8. FAB$^+$ mass spectrum of ligand **L2,** 2,2'-(ethane-1,2-diylbis(oxy))bis(N-benzylethan-1-amine) recorded in acetonitrile.

Compounds [Ni(N_2O_2)(NO_3)$_2$] and [Ni(N_2O_2)(O–O)]NO_3

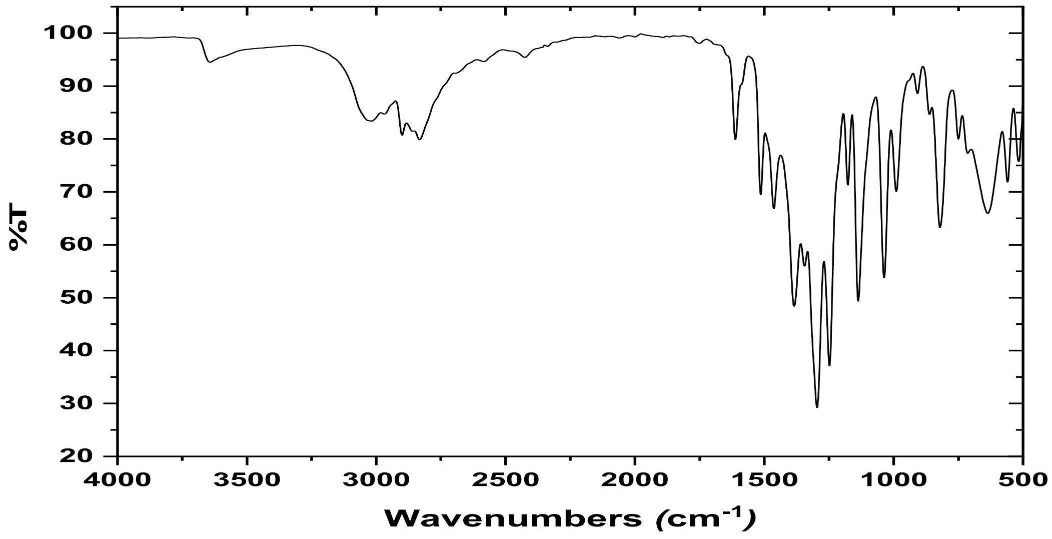

Figure S9. Infrared spectrum (400–4000 cm^{-1}) of the complex [Ni(L1)(NO_3)$_2$], **1a**

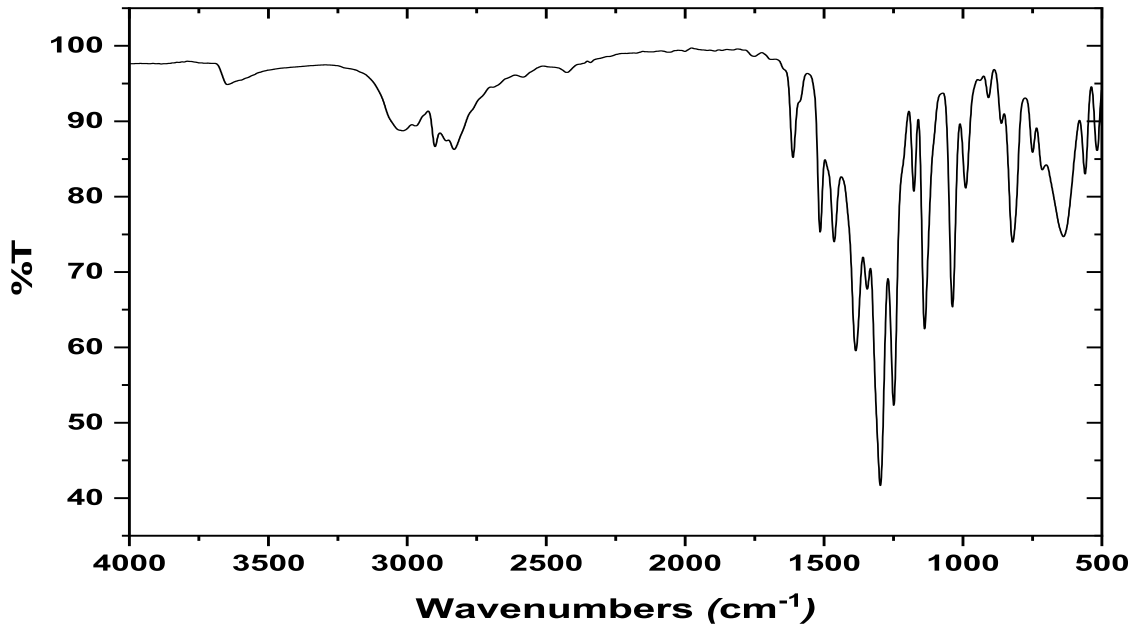

Figure S10. Infrared spectrum (400–4000 cm^{-1}) of the complex [Ni(L1)(acac)]NO_3, **2a**

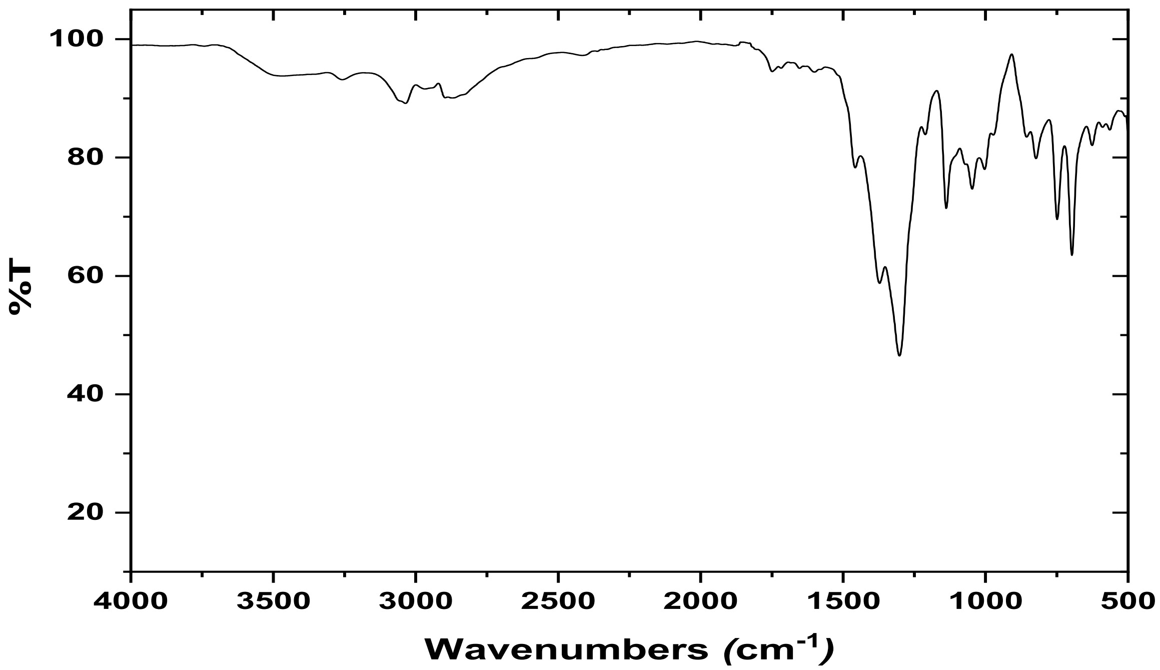

Figure S11. Infrared spectrum (400–4000 cm^{-1}) of the complex [Ni(L1)(tfac)]NO_3, **3a**

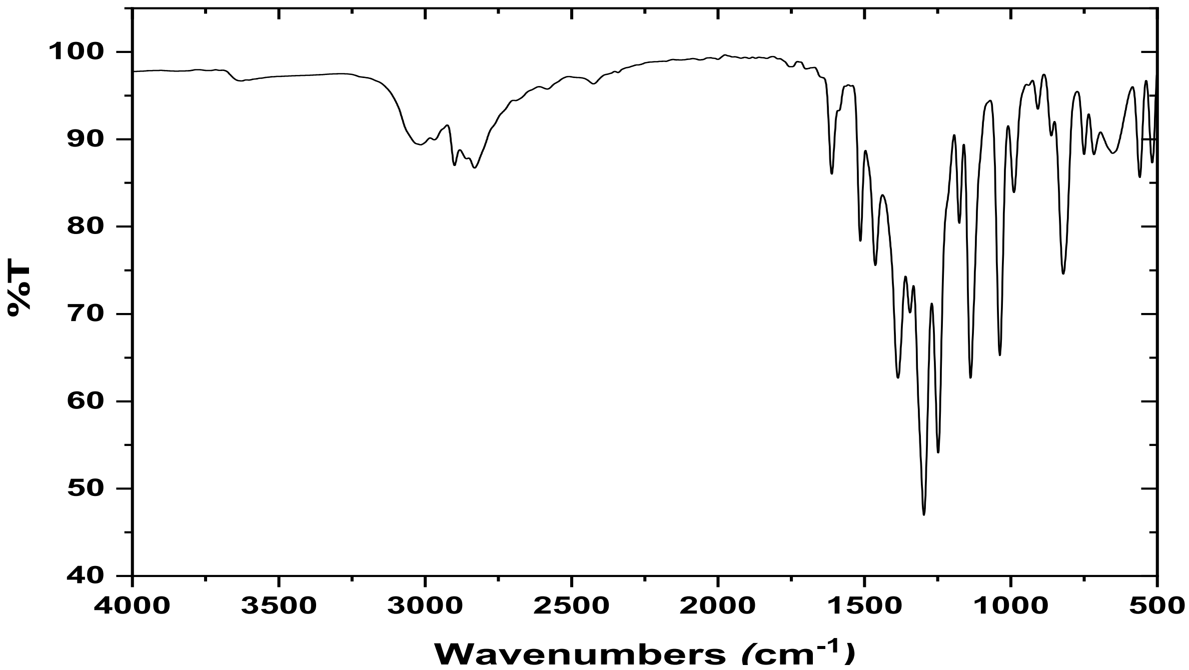

Figure S12. Infrared spectrum (400–4000 cm^{-1}) of the complex $[Ni(L1)(hfac)]NO_3$, **4a**

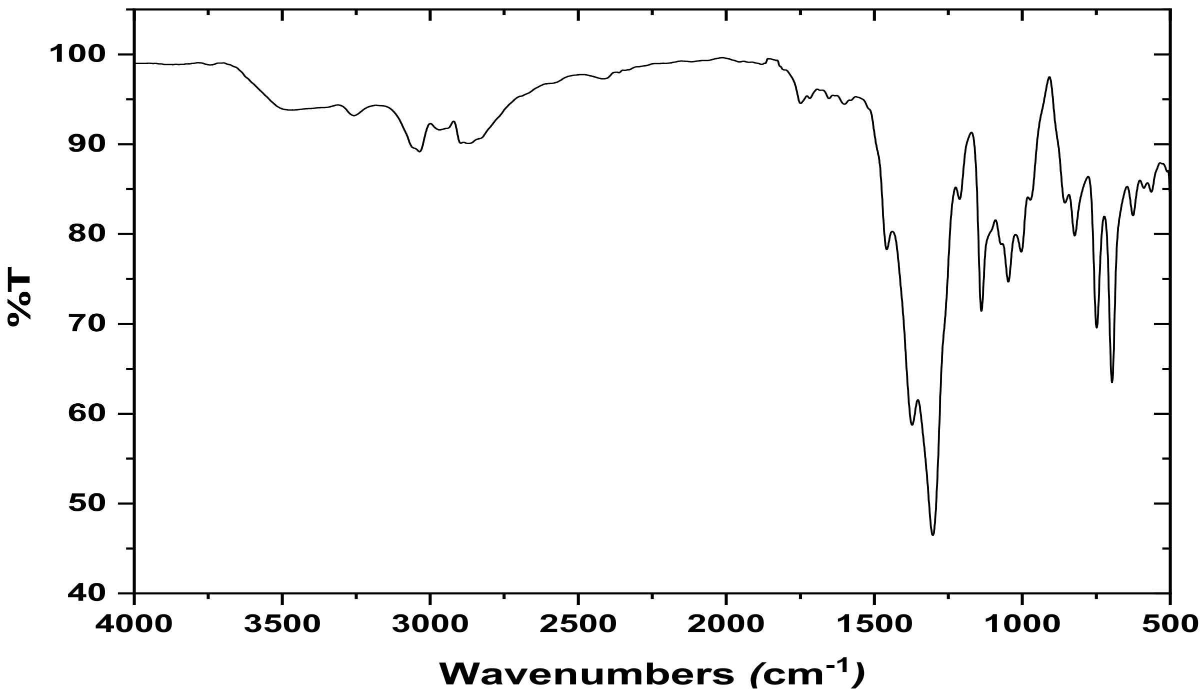

Figure S13. Infrared spectrum (400–4000 cm^{-1}) of the complex $[Ni(L2)(NO_3)_2]$, **1b**

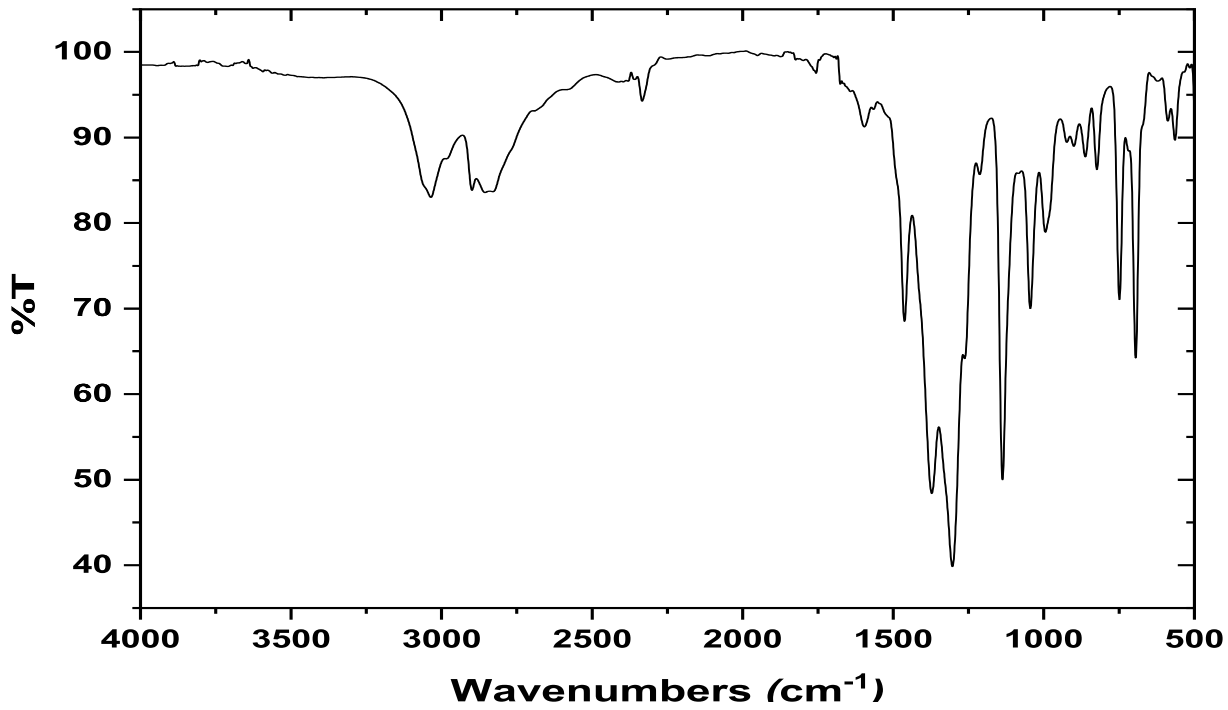

Figure S14. Infrared spectrum (400–4000 cm^{-1}) of the complex [Ni(L2)(acac)]NO_3, **2b**

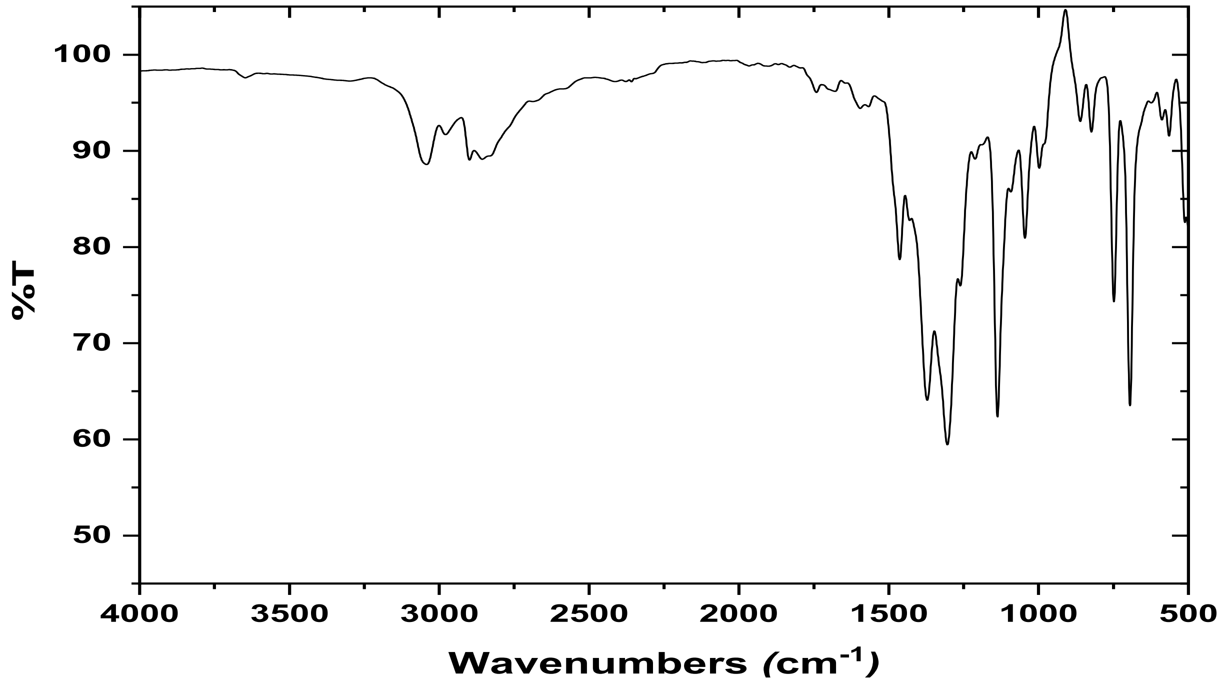

Figure S15. Infrared spectrum (400–4000 cm^{-1}) of the complex [Ni(L2)(tfac)]NO_3, **3b**

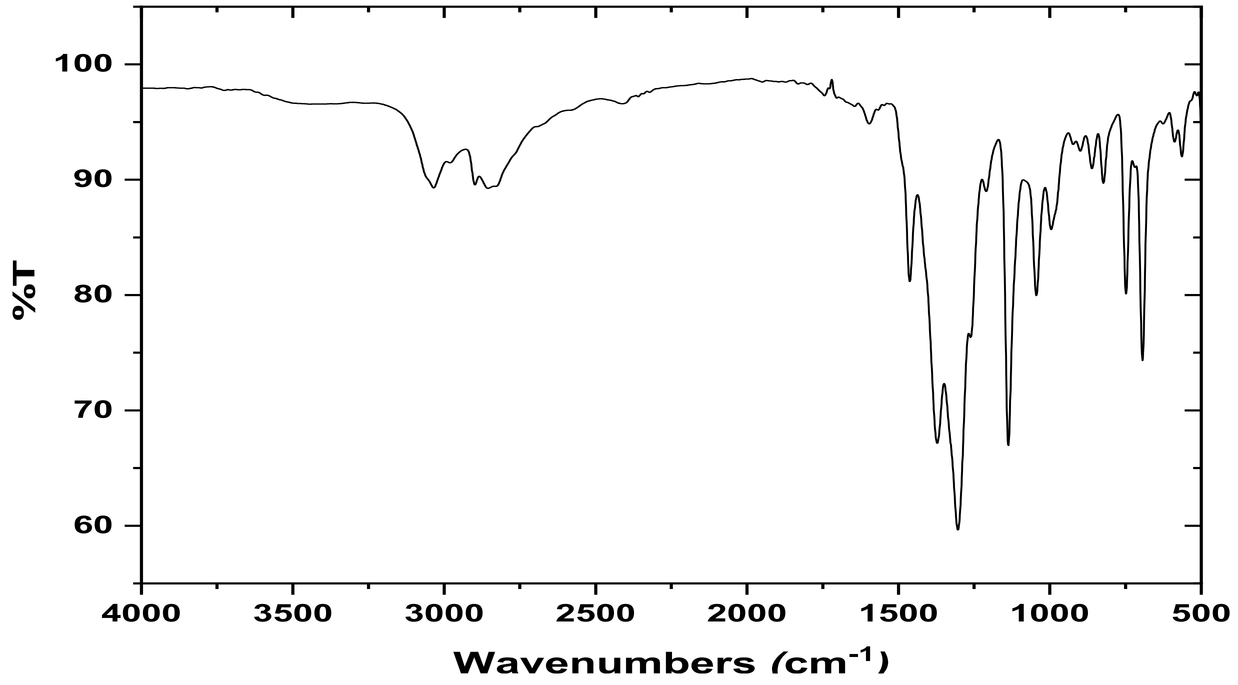

Figure S16. Infrared spectrum (400–4000 cm^{-1}) of the complex [Ni(L2)(hfac)]NO_3, **4b**

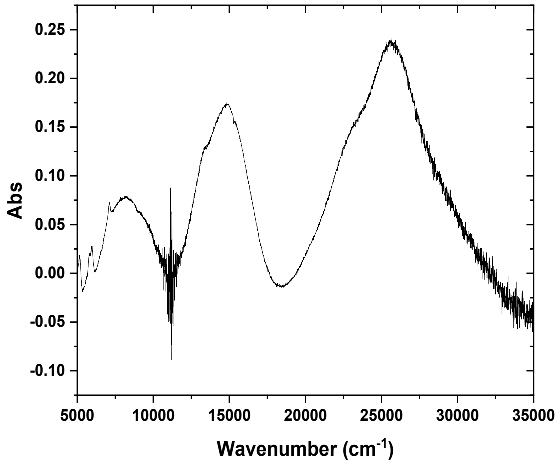

Figure S17. Diffuse reflectance spectrum (5000–35000 cm^{-1}) of the complex [Ni(L1)(NO_3)$_2$] **1a**

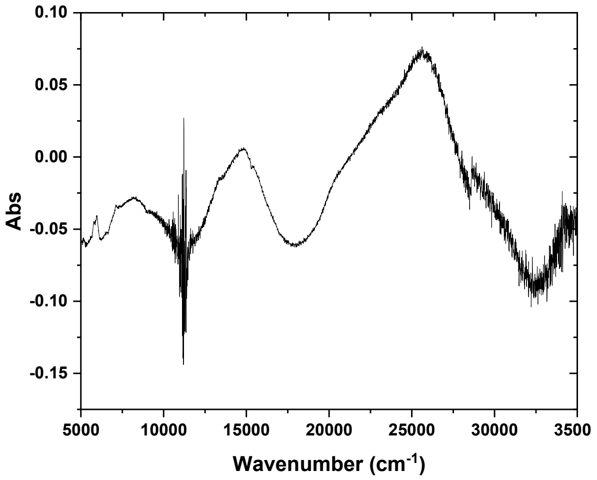

Figure S18. Diffuse reflectance spectrum (5000–35000 cm^{-1}) of the complex [Ni(L1)(acac)]NO_3, **2a**

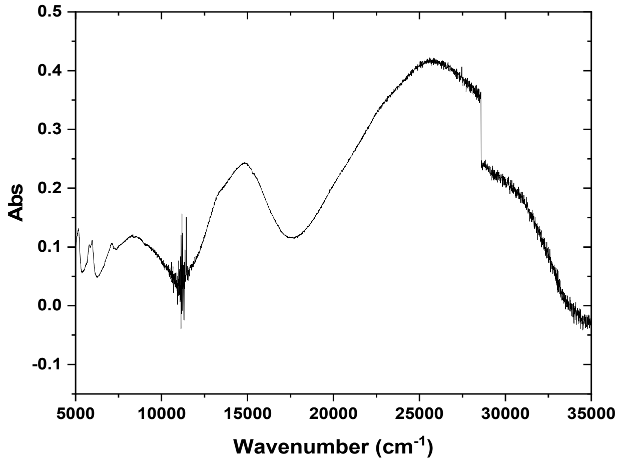

Figure S19. Diffuse reflectance spectrum (5000–35000 cm^{-1}) of the complex $[Ni(L1)(tfac)]NO_3$, **3a**

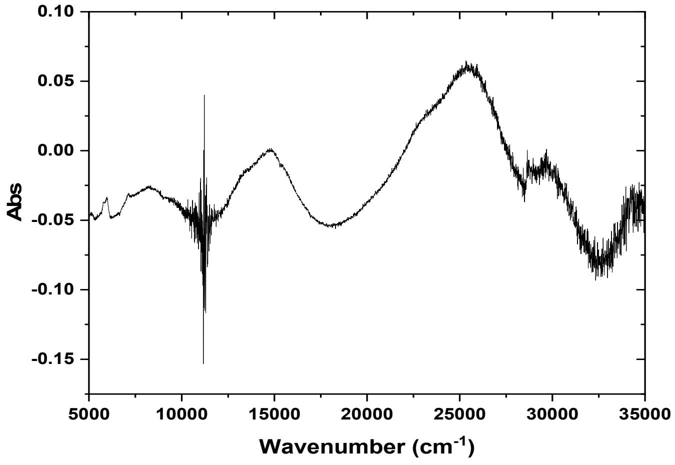

Figure S20. Diffuse reflectance spectrum (5000–35000 cm^{-1}) of the complex $[Ni(L1)(hfac)]NO_3$, **4a**

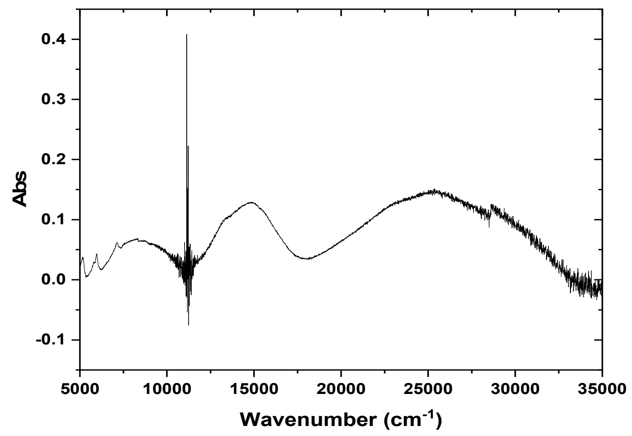

Figure S21. Diffuse reflectance spectrum (5000–35000 cm^{-1}) of the complex $[Ni(L2)(NO_3)_2]$, **1b**

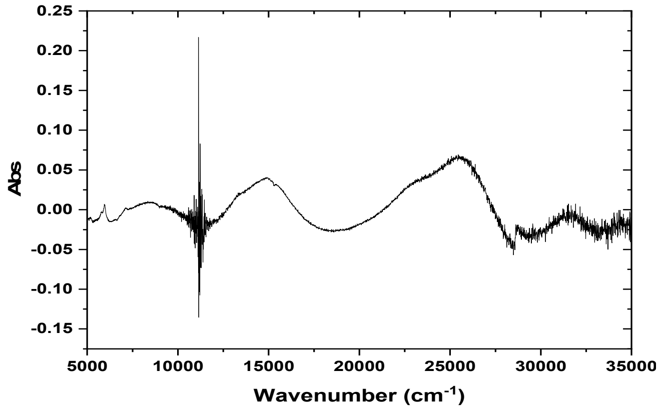

Figure S22. Diffuse reflectance spectrum (5000–35000 cm^{-1}) of the complex $[Ni(L2)(acac)]NO_3$, **2b**

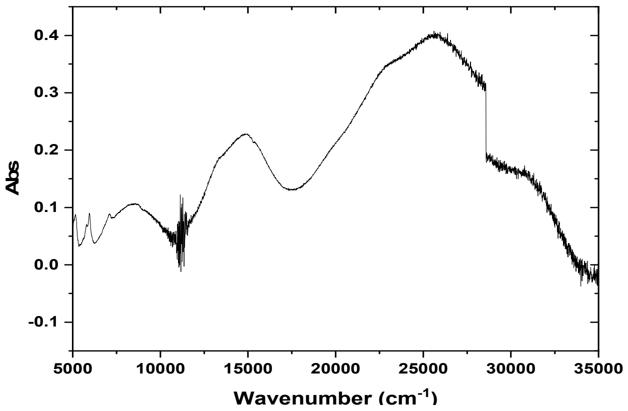

Figure S23. Diffuse reflectance spectrum (5000–35000 cm^{-1}) of the complex [Ni(L2)(tfac)]NO_3, **3b**

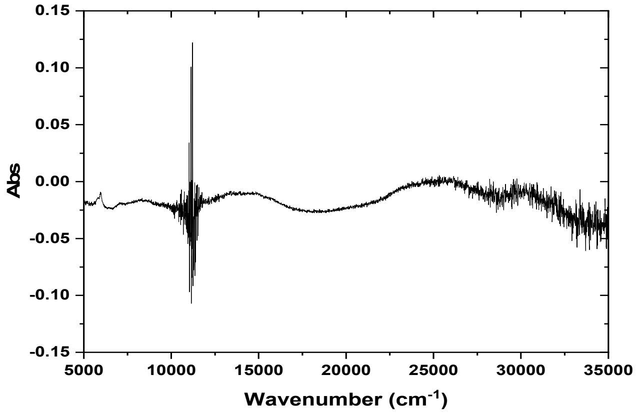

Figure S24. Diffuse reflectance spectrum (5000–35000 cm^{-1}) of the complex [Ni(L2)(hfac)]NO_3, **4b**

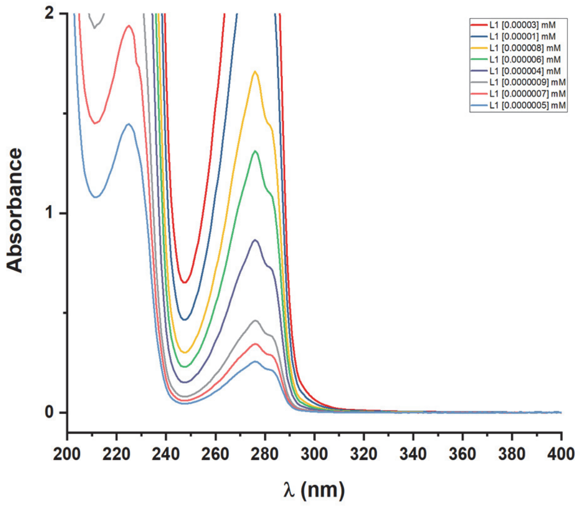

Figure S25. UV–Vis absorption spectra of ligand **L1** recorded in CH_3CN at different concentrations (3.0 × 10^{-5}–5.0 × 10^{-7} mM) over the wavelength range of 200–400 nm

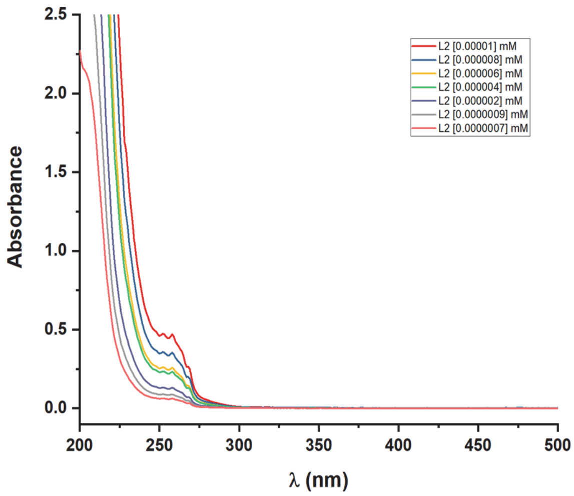

Figure S26. UV–Vis absorption spectra of ligand **L2** recorded in CH_3CN at different concentrations (1.0 × 10^{-5}–7.0 × 10^{-7} mM) over the wavelength range of 200–500 nm

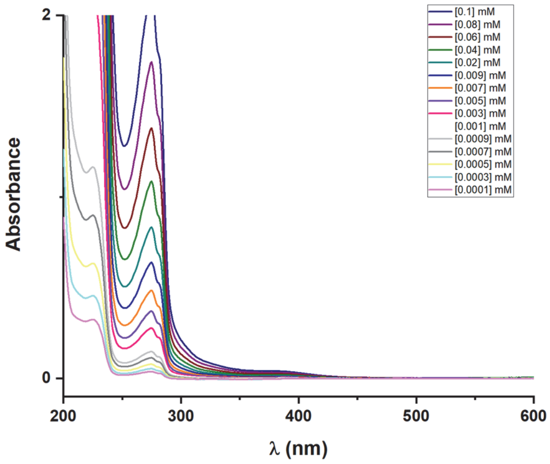

Figure S27. UV–Vis absorption spectra of complex **1a**, $[Ni(L1)(NO_3)_2]$, recorded in CH_3CN at different concentrations (1.1×10^{-1}–1.0×10^{-4} mM) over the wavelength range of 200–600 nm.

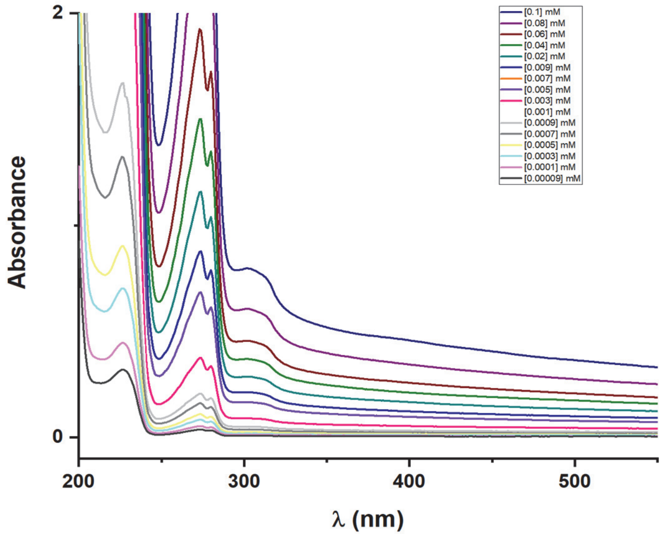

Figure S28. UV–Vis absorption spectra of complex **2a**, $[Ni(L1)(acac)]NO_3$, recorded in CH_3CN at different concentrations (1.0×10^{-1}–1.0×10^{-4} mM) over the wavelength range of 200–550 nm.

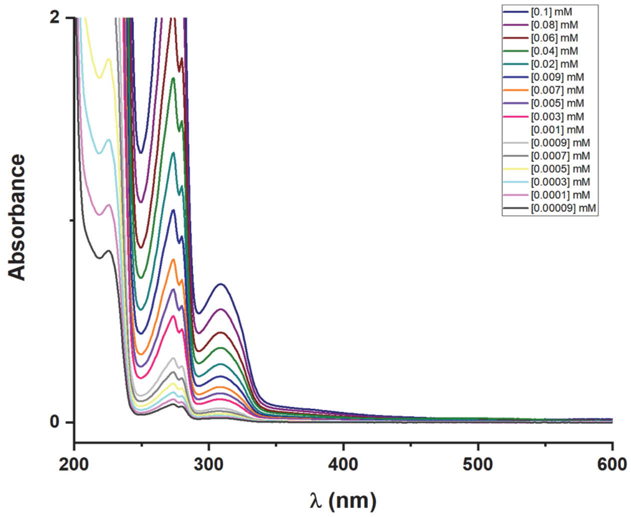

Figure S29. UV–Vis absorption spectra of complex **3a,** $[Ni(L1)(tfac)]NO_3$, recorded in CH_3CN at different concentrations (1.0×10^{-1}–9.0×10^{-5} mM) over the wavelength range of 200–600 nm.

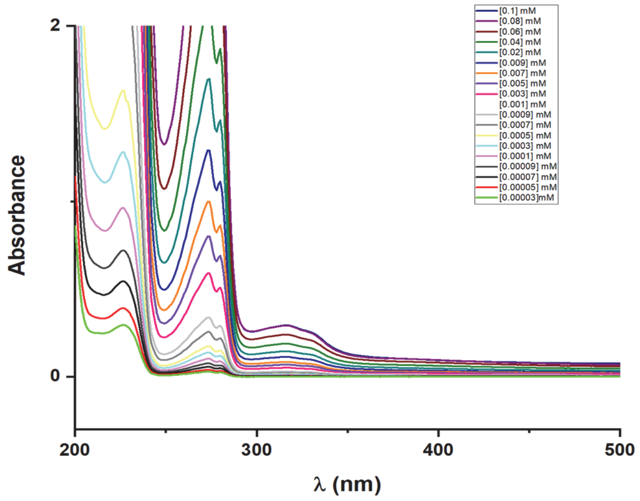

Figure S30. UV–Vis absorption spectra of complex **4a**, $[Ni(L1)(hfac)]NO_3$, recorded in CH_3CN at different concentrations (1.0×10^{-1}–3.0×10^{-5} mM) over the wavelength range of 200–500 nm.

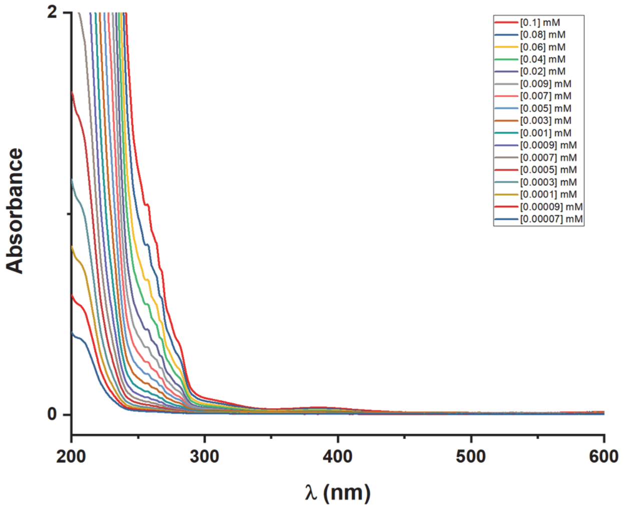

Figure S31. UV–Vis absorption spectra of complex **1b**, $[Ni(L2)(NO_3)_2]$, recorded in CH_3CN at different concentrations (1.1×10^{-1}–7.0×10^{-5} mM) over the wavelength range of 200–600 nm.

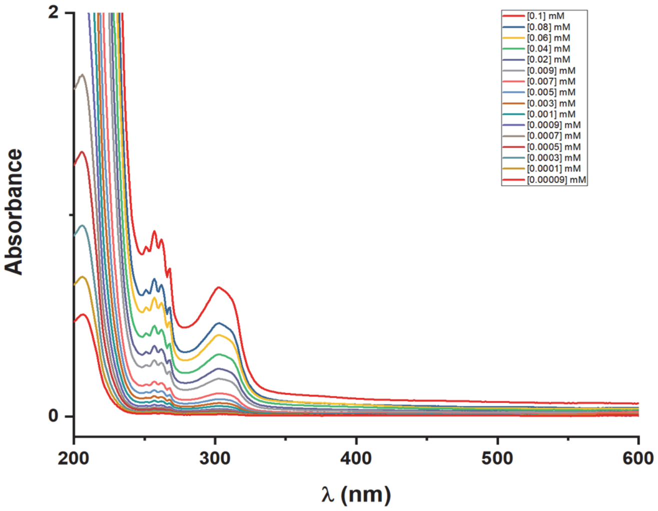

Figure S32. UV–Vis absorption spectra of complex **2b**, $[Ni(L2)(acac)]NO_3$, recorded in CH_3CN at different concentrations (1.0×10^{-1}–9.0×10^{-5} mM) over the wavelength range of 200–600 nm.

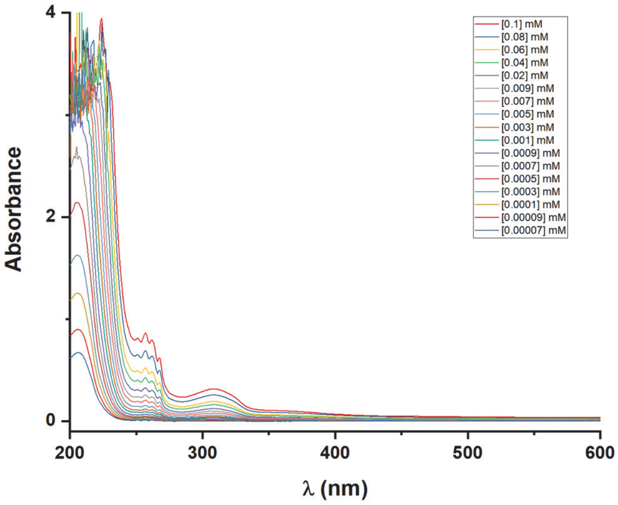

Figure S33. UV–Vis absorption spectra of complex **3b**, $[Ni(L2)(tfac)]NO_3$, recorded in CH_3CN at different concentrations over the wavelength range of 200–600 nm

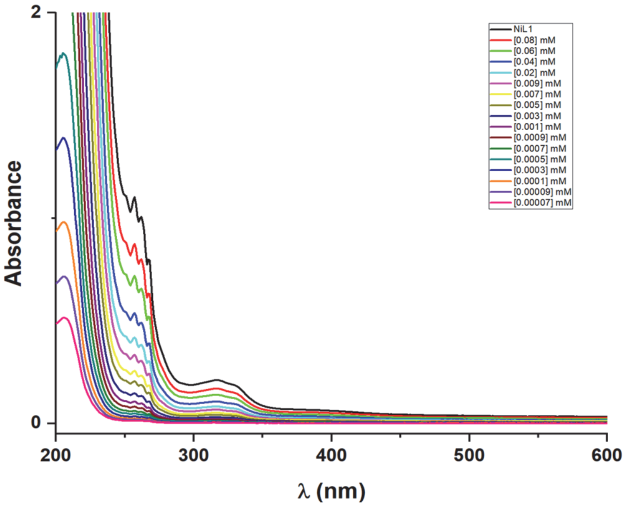

Figure S34. UV–Vis absorption spectra of compound $[Ni(L2)(hfac)]$, **4b** recorded in acetonitrile at different concentrations (8.0×10^{-2}–7.0×10^{-4} mM) over the wavelength range of 200–600 nm.

Table S1. Electronic ultraviolet-visible spectra data in CH_3CN (nm); in parentheses, the DFT calculated (UB3LYP/GENECP) are presented. For comparison purposes diffuse reflectance values were transformed (cm^{-1} to nm)

Code	λ (π-π*) (N_2O_2) (nm)	λ (π-π*) (N_2O_2) (nm)	λ (π-π*) β diketone (nm)	λ MLCT (nm)	$^3A_2g \rightarrow {}^3T_2g(F)$ (nm)	$^3A_2g \rightarrow {}^3T_1g(F)$ (nm)	$^3A_2g \rightarrow {}^3T_1g(P)$ (nm)
**1a**	225	274	-	383	1221	673	390
	(229)	(275)		(385)	(N.D.)	(741)	(385)
**2a**	225	273	316	-	1218	674	389
	(227)	(267)	(321)		(N.D.)	(734)	(402)
**3a**	226	273	310	-	1192	672	388
	(227)	(270)	(314)		(N.D.)	(725)	(429)
**4a**	226	274	308	-	1205	675	394
	(227)	(295)	(330)		(N.D.)	(744)	(396)
**1b**	207	257	-	386	1193	674	394
	(212)	(272)		(411)	(N.D)	(744)	(411)
**2b**	206	257	313	-	1186	668	393
	(211)	(256)	(322)		(N.D.)	(736)	(402)
**3b**	205	256	308	-	1176	671	389
	(209)	(264)	(306)		(N.D.)	(727)	(423)
**4b**	205	257	306	-	1185	681	391
	(211)	(253)	(296)		(N.D.)	(733)	(422)

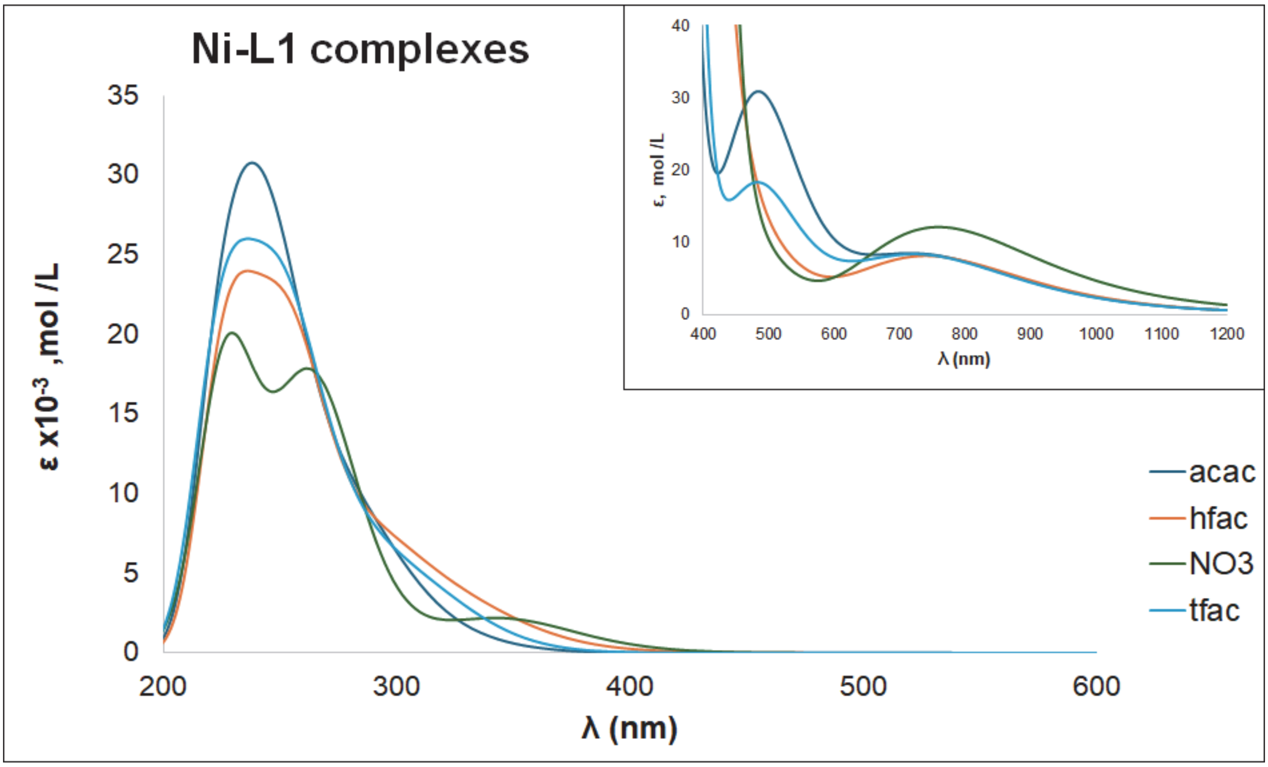

Figure S35. Calculated (UB3LYP/GENECP) UV–Vis absorption spectra of Ni-L1 complexes.

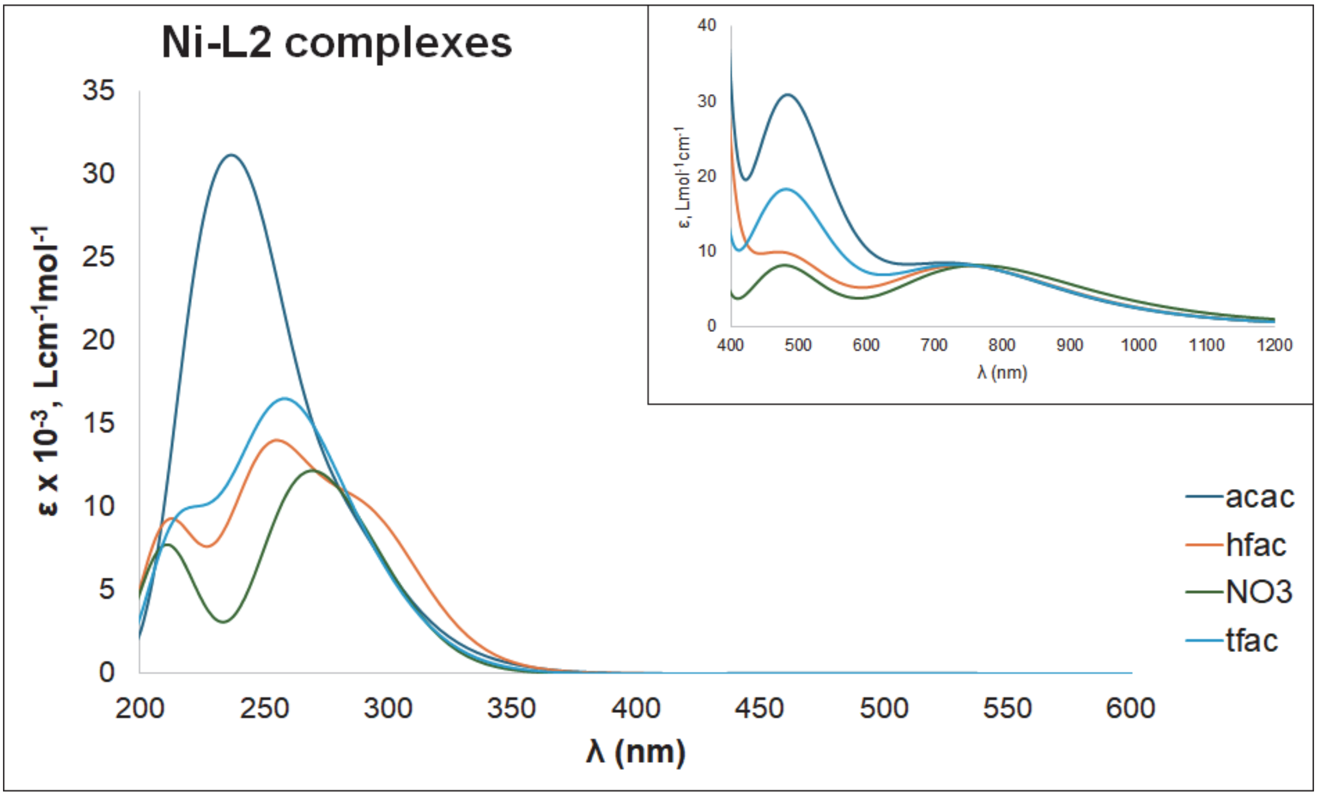

Figure S36. Calculated (UB3LYP/GENECP) UV–Vis absorption spectra of Ni-L2 complexes.

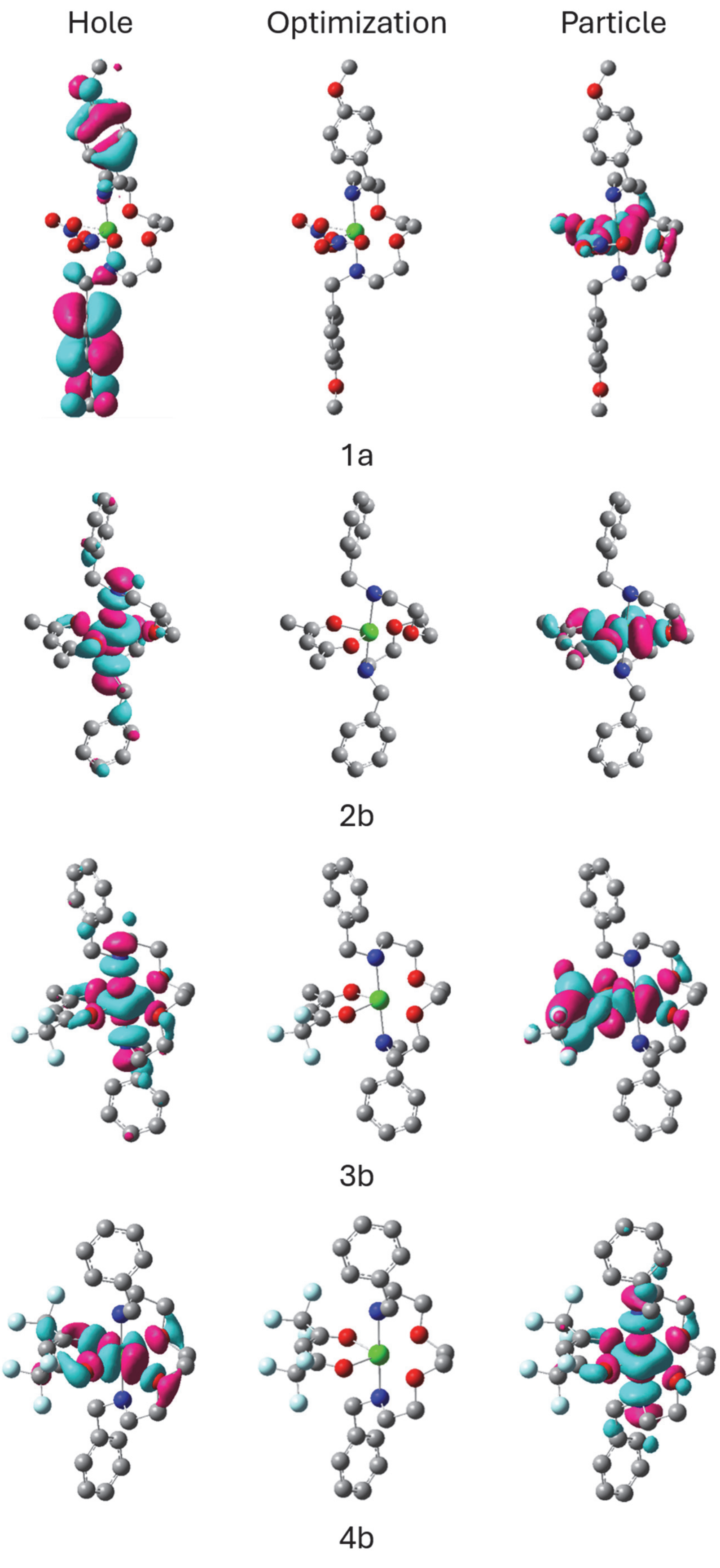

Figure S37. NTO for compounds 1a and 2b-4b

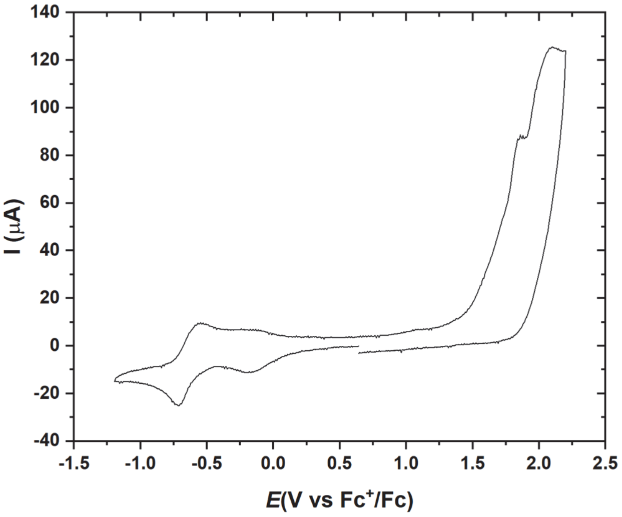

Figure S38. Cyclic voltammogram of **1a** [NiL1]NO_3 (2.0 mM) recorded in CH_3CN containing 0.1 M $TBAPF_6$ using a Pt working electrode at a scan rate of 300 mV s^{-1}.

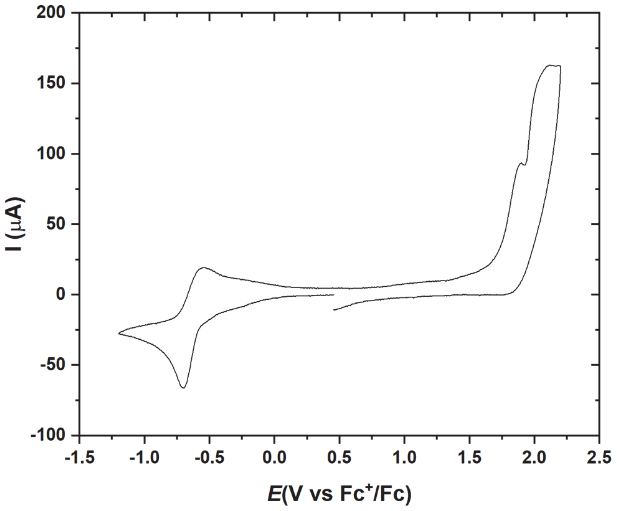

Figure S39. Cyclic voltammogram of **2a** [Ni(L1)(acac)]NO_3 (2.0 mM) recorded in CH_3CN containing 0.1 M $TBAPF_6$ using a Pt working electrode at a scan rate of 300 mV s^{-1}.

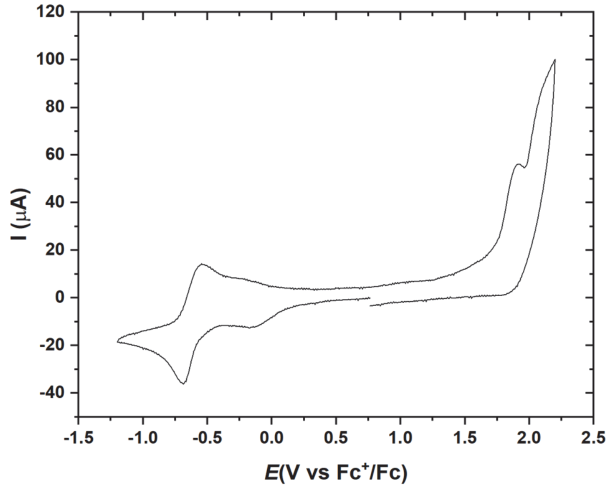

Figure S40. Cyclic voltammogram of **3a** [Ni(L1)(tfac)]NO_3 (2.0 mM) recorded in CH_3CN containing 0.1 M $TBAPF_6$ using a Pt working electrode at a scan rate of 300 mV s^{-1}.

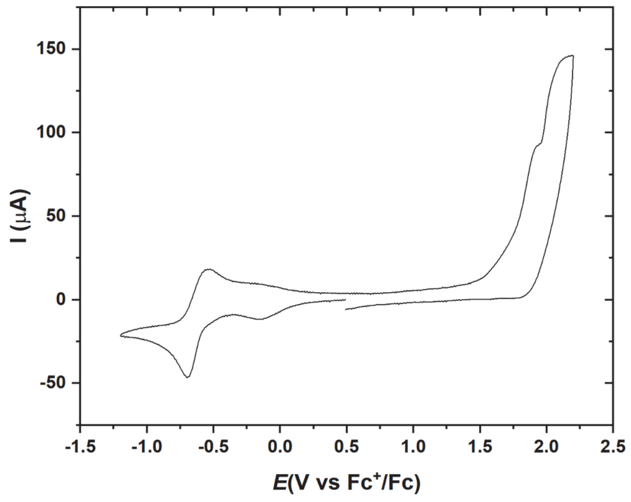

Figure S41. Cyclic voltammogram of 4a [Ni(L1)(hfac)]NO_3 (2.0 mM) recorded in CH_3CN containing 0.1 M $TBAPF_6$ using a Pt working electrode at a scan rate of 300 mV s^{-1}.

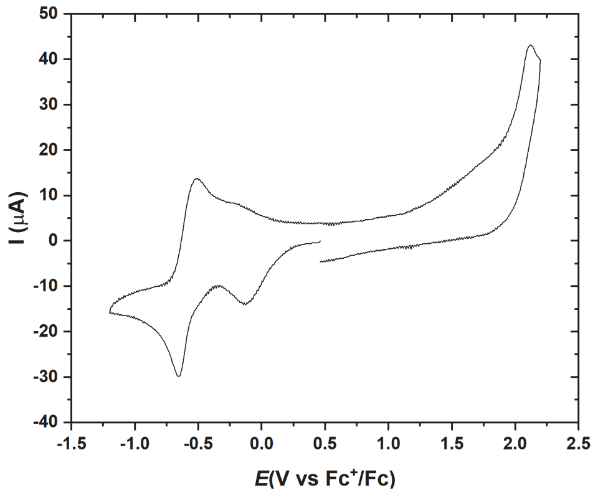

Figure S42. Cyclic voltammogram of **1b** [NiL2]NO_3 (2.0 mM) recorded in CH_3CN containing 0.1 M $TBAPF_6$ using a Pt working electrode at a scan rate of 300 mV s^{-1}.

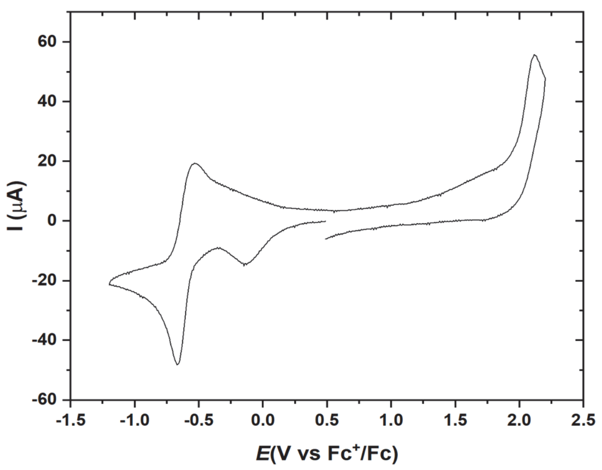

Figure S43. Cyclic voltammogram of **2b** [Ni(L2)(acac)]NO_3 (2.0 mM) recorded in CH_3CN containing 0.1 M $TBAPF_6$ using a Pt working electrode at a scan rate of 300 mV s^{-1}.

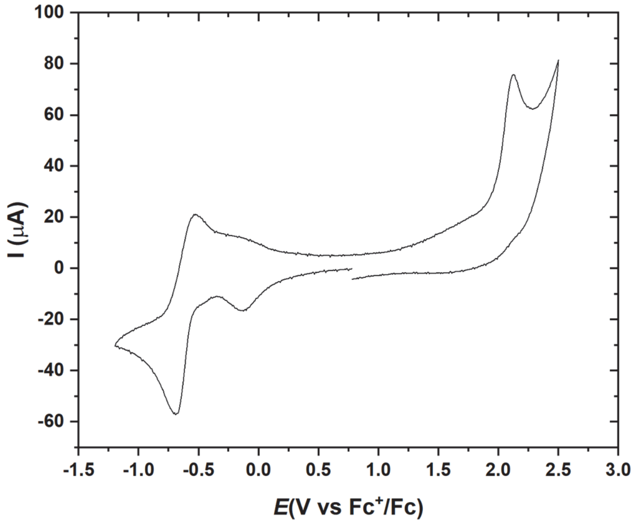

Figure S44. Cyclic voltammogram of **3b** [Ni(L2)(tfac)]NO_3 (2.0 mM) recorded in CH_3CN containing 0.1 M $TBAPF_6$ using a Pt working electrode at a scan rate of 300 mV s^{-1}.

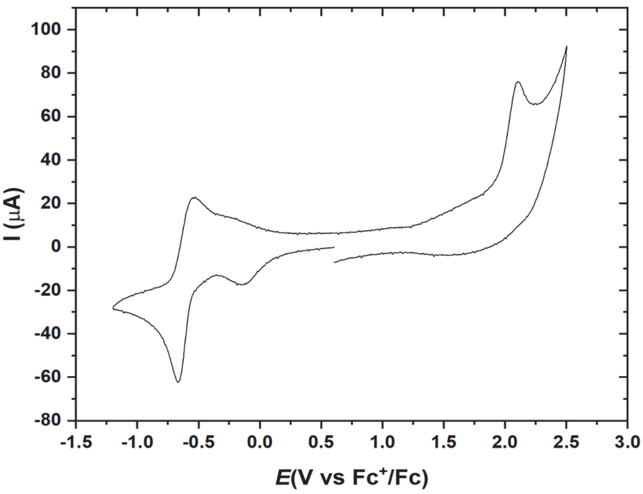

Figure S45. Cyclic voltammogram of **4b** [Ni(L1)(hfac)]NO_3 (2.0 mM) recorded in CH_3CN containing 0.1 M $TBAPF_6$ using a Pt working electrode at a scan rate of 300 mV s^{-1}.

Biological activity

Table S2. Individual IC_{50} values obtained from three independent biological experiments, corresponding coefficients of determination (R^2) for the dose–response curve fitting, and the calculated mean ± standard deviation (SD) for compounds **1a–4a**.

Compound	Parameter	Experiment 1	Experiment 2	Experiment 3	Mean	SD
**1a**	IC_{50} (μM)	144.86357	111.30731	162.99206	**139.7**	26.2
	R^2	0.9161	0.94881	0.98671		
**2a**	IC_{50} (μM)	76.79416	67.95926	94.11685	**79.6**	13.3
	R^2	0.99958	0.9885	0.989		
**3a**	IC_{50} (μM)	62.72483	76.9744	75.92666	**71.9**	7.9
	R^2	0.997	0.99539	0.99702		
**4a**	IC_{50} (μM)	52.0058	65.38491	50.56238	**56.0**	8.2
	R^2	0.99732	0.9986	0.98392		

Crystallographic data

Table S3. Atomic coordinates (x 10^4) and equivalent isotropic displacement parameters (Å^{2}x 10^3) for nil1acf6-143. U(eq) is defined as one third of the trace of the orthogonalized U^{ij} tensor.

	x	y	z	U(eq)
N(1)	3212(1)	2727(2)	6562(1)	21(1)
N(2)	3176(1)	7742(2)	4967(1)	24(1)
O(2)	4506(1)	3962(2)	6134(1)	24(1)
O(3)	3093(1)	6020(2)	4326(1)	37(1)
O(4)	3234(1)	7558(2)	6090(1)	28(1)
O(5)	3210(1)	9726(2)	4518(1)	41(1)
C(7)	2561(1)	2428(3)	7094(2)	25(1)
C(8)	3804(1)	2048(3)	7393(2)	24(1)
C(9)	4409(1)	1810(3)	6704(2)	26(1)
C(10)	4987(1)	3826(3)	5289(2)	24(1)
C(1)	2007(5)	3377(17)	6203(7)	23(2)
O(1)	528(2)	5990(6)	3680(3)	68(1)
C(2)	1694(2)	5469(8)	6410(4)	36(1)
C(3)	1200(3)	6428(12)	5606(7)	43(2)
C(4)	1012(2)	5253(11)	4554(5)	42(1)
C(5)	1314(2)	3158(9)	4316(4)	42(1)
C(6)	1795(3)	2220(9)	5147(5)	31(1)
C(11)	181(5)	8072(12)	3858(9)	81(2)
C(1P)	1993(8)	3740(30)	6479(11)	23(2)

O(1P)	482(3)	7723(9)	4736(5)	68(1)
C(2P)	1768(4)	5749(12)	6959(7)	36(2)
C(3P)	1260(4)	7026(11)	6373(8)	51(2)
C(4P)	998(4)	6294(18)	5246(10)	36(2)
C(5P)	1216(4)	4351(14)	4715(7)	36(2)
C(6P)	1728(5)	3059(14)	5350(8)	34(2)
C(11P)	232(9)	7160(20)	3569(15)	81(2)

Table S4. Bond lengths [Å] and angles [°] for nil1acf6-143.

N(1)-C(8)	1.490(2)
N(1)-C(7)	1.499(2)
N(1)-H(1E)	0.95(2)
N(1)-H(1D)	0.95(2)
N(2)-O(3)	1.2304(19)
N(2)-O(4)	1.2564(18)
N(2)-O(5)	1.2578(18)
O(2)-C(10)	1.4204(19)
O(2)-C(9)	1.421(2)
C(7)-C(1P)	1.484(10)
C(7)-C(1)	1.522(6)
C(7)-H(7A)	0.9900
C(7)-H(7B)	0.9900
C(8)-C(9)	1.510(2)
C(8)-H(8A)	0.9900
C(8)-H(8B)	0.9900
C(9)-H(9A)	0.9900
C(9)-H(9B)	0.9900
C(10)-C(10)#1	1.508(3)
C(10)-H(10A)	0.9900
C(10)-H(10B)	0.9900
C(1)-C(6)	1.390(7)
C(1)-C(2)	1.394(7)
O(1)-C(4)	1.379(5)
O(1)-C(11)	1.416(6)
C(2)-C(3)	1.389(7)
C(2)-H(2)	0.9500
C(3)-C(4)	1.381(8)

C(3)-H(3)	0.9500
C(4)-C(5)	1.393(6)
C(5)-C(6)	1.386(6)
C(5)-H(5)	0.9500
C(6)-H(6)	0.9500
C(11)-H(11A)	0.9800
C(11)-H(11B)	0.9800
C(11)-H(11C)	0.9800
C(1P)-C(2P)	1.375(10)
C(1P)-C(6P)	1.381(10)
O(1P)-C(11P)	1.392(14)
O(1P)-C(4P)	1.403(10)
C(2P)-C(3P)	1.374(9)
C(2P)-H(2P)	0.9500
C(3P)-C(4P)	1.386(10)
C(3P)-H(3P)	0.9500
C(4P)-C(5P)	1.364(13)
C(5P)-C(6P)	1.407(10)
C(5P)-H(5P)	0.9500
C(6P)-H(6P)	0.9500
C(11P)-H(11D)	0.9800
C(11P)-H(11E)	0.9800
C(11P)-H(11F)	0.9800
C(8)-N(1)-C(7)	113.25(13)
C(8)-N(1)-H(1E)	108.8(11)
C(7)-N(1)-H(1E)	107.3(11)
C(8)-N(1)-H(1D)	111.4(11)
C(7)-N(1)-H(1D)	106.4(11)
H(1E)-N(1)-H(1D)	109.6(16)
O(3)-N(2)-O(4)	120.69(14)
O(3)-N(2)-O(5)	120.97(15)
O(4)-N(2)-O(5)	118.34(14)
C(10)-O(2)-C(9)	112.51(12)
C(1P)-C(7)-N(1)	114.7(8)
N(1)-C(7)-C(1)	107.9(5)
N(1)-C(7)-H(7A)	110.1
C(1)-C(7)-H(7A)	110.1
N(1)-C(7)-H(7B)	110.1

C(1)-C(7)-H(7B)	110.1
H(7A)-C(7)-H(7B)	108.4
N(1)-C(8)-C(9)	109.77(13)
N(1)-C(8)-H(8A)	109.7
C(9)-C(8)-H(8A)	109.7
N(1)-C(8)-H(8B)	109.7
C(9)-C(8)-H(8B)	109.7
H(8A)-C(8)-H(8B)	108.2
O(2)-C(9)-C(8)	107.57(13)
O(2)-C(9)-H(9A)	110.2
C(8)-C(9)-H(9A)	110.2
O(2)-C(9)-H(9B)	110.2
C(8)-C(9)-H(9B)	110.2
H(9A)-C(9)-H(9B)	108.5
O(2)-C(10)-C(10)#1	106.92(17)
O(2)-C(10)-H(10A)	110.3
C(10)#1-C(10)-H(10A)	110.3
O(2)-C(10)-H(10B)	110.3
C(10)#1-C(10)-H(10B)	110.3
H(10A)-C(10)-H(10B)	108.6
C(6)-C(1)-C(2)	117.0(5)
C(6)-C(1)-C(7)	121.9(5)
C(2)-C(1)-C(7)	121.2(5)
C(4)-O(1)-C(11)	119.1(5)
C(3)-C(2)-C(1)	123.0(5)
C(3)-C(2)-H(2)	118.5
C(1)-C(2)-H(2)	118.5
C(4)-C(3)-C(2)	118.3(5)
C(4)-C(3)-H(3)	120.8
C(2)-C(3)-H(3)	120.8
O(1)-C(4)-C(3)	124.7(5)
O(1)-C(4)-C(5)	115.0(5)
C(3)-C(4)-C(5)	120.3(4)
C(6)-C(5)-C(4)	120.0(4)
C(6)-C(5)-H(5)	120.0
C(4)-C(5)-H(5)	120.0
C(5)-C(6)-C(1)	121.3(5)
C(5)-C(6)-H(6)	119.4
C(1)-C(6)-H(6)	119.4

O(1)-C(11)-H(11A)	109.5
O(1)-C(11)-H(11B)	109.5
H(11A)-C(11)-H(11B)	109.5
O(1)-C(11)-H(11C)	109.5
H(11A)-C(11)-H(11C)	109.5
H(11B)-C(11)-H(11C)	109.5
C(2P)-C(1P)-C(6P)	119.2(8)
C(2P)-C(1P)-C(7)	121.2(8)
C(6P)-C(1P)-C(7)	119.3(7)
C(11P)-O(1P)-C(4P)	115.4(8)
C(3P)-C(2P)-C(1P)	121.6(8)
C(3P)-C(2P)-H(2P)	119.2
C(1P)-C(2P)-H(2P)	119.2
C(2P)-C(3P)-C(4P)	118.1(7)
C(2P)-C(3P)-H(3P)	121.0
C(4P)-C(3P)-H(3P)	121.0
C(5P)-C(4P)-C(3P)	122.6(8)
C(5P)-C(4P)-O(1P)	124.3(9)
C(3P)-C(4P)-O(1P)	113.0(9)
C(4P)-C(5P)-C(6P)	117.9(7)
C(4P)-C(5P)-H(5P)	121.1
C(6P)-C(5P)-H(5P)	121.1
C(1P)-C(6P)-C(5P)	120.6(8)
C(1P)-C(6P)-H(6P)	119.7
C(5P)-C(6P)-H(6P)	119.7
O(1P)-C(11P)-H(11D)	109.5
O(1P)-C(11P)-H(11E)	109.5
H(11D)-C(11P)-H(11E)	109.5
O(1P)-C(11P)-H(11F)	109.5
H(11D)-C(11P)-H(11F)	109.5
H(11E)-C(11P)-H(11F)	109.5

Symmetry transformations used to generate equivalent atoms:

#1 -x+1,-y+1,-z+1

Table S5. Anisotropic displacement parameters ($Å^2$x 10^3)for nil1acf6-143. The anisotropic displacement factor exponent takes the form: $-2\pi^2[h^2a^{*2}U^{11} + ... + 2 h k a^* b^* U^{12}]$

	U^{11}	U^{22}	U^{33}	U^{23}	U^{13}	U^{12}
N(1)	28(1)	17(1)	17(1)	1(1)	3(1)	0(1)
N(2)	32(1)	21(1)	18(1)	-1(1)	4(1)	-1(1)
O(2)	28(1)	23(1)	24(1)	1(1)	7(1)	-1(1)
O(3)	61(1)	26(1)	25(1)	-10(1)	7(1)	-4(1)
O(4)	47(1)	22(1)	15(1)	1(1)	5(1)	-1(1)
O(5)	79(1)	23(1)	21(1)	6(1)	3(1)	-4(1)
C(7)	28(1)	27(1)	21(1)	-1(1)	6(1)	-1(1)
C(8)	28(1)	26(1)	16(1)	2(1)	1(1)	-1(1)
C(9)	28(1)	26(1)	23(1)	4(1)	1(1)	2(1)
C(10)	23(1)	28(1)	21(1)	-1(1)	4(1)	1(1)
C(1)	24(2)	24(3)	23(4)	-4(2)	9(3)	-1(2)
O(1)	57(2)	70(2)	69(2)	-2(1)	-26(1)	18(1)
C(2)	35(2)	34(2)	38(3)	-14(2)	-1(2)	0(2)
C(3)	34(3)	33(2)	59(5)	-8(3)	-10(2)	6(2)
C(4)	34(3)	45(3)	44(3)	5(2)	-6(2)	6(2)
C(5)	31(2)	60(3)	32(2)	-14(2)	-2(2)	7(2)
C(6)	26(2)	34(3)	31(2)	-7(2)	0(2)	3(2)
C(11)	55(3)	65(5)	117(6)	29(5)	-30(3)	22(4)
C(1P)	30(4)	22(5)	18(5)	0(3)	12(4)	-2(3)
O(1P)	57(2)	70(2)	69(2)	-2(1)	-26(1)	18(1)
C(2P)	36(3)	26(3)	44(4)	-5(3)	-3(4)	5(2)
C(3P)	63(5)	29(3)	56(5)	-3(3)	-16(4)	8(3)
C(4P)	28(5)	35(4)	45(6)	8(4)	-8(4)	-8(4)
C(5P)	44(5)	37(5)	27(4)	7(3)	-1(3)	-5(4)
C(6P)	33(4)	26(4)	43(5)	-4(3)	0(3)	-1(3)
C(11P)	55(3)	65(5)	117(6)	29(5)	-30(3)	22(4)
